# Hemoglobin β-F41K: A recombinant oxygen carrier prototype engineered for enhanced heme retention, stability, and optimal oxygenation properties

**DOI:** 10.1016/j.jbc.2025.110477

**Published:** 2025-07-11

**Authors:** Mohd Asim Khan, Kajal Yadav, Anthony V. Signore, Nidhi Mittal, Sheetal Uppal, Amit K. Tyagi, Gaurav Mittal, Jay F. Storz, Suman Kundu

**Affiliations:** 1 Department of Biochemistry, University of Delhi South Campus, New Delhi, India; 2 School of Biological Sciences, University of Nebraska, Lincoln, Nebraska, USA; 3 Institute of Nuclear Medicine and Allied Sciences, Defence Research and Development Organization, Delhi, India; 4 Department of Biological Sciences, Birla Institute of Technology and Science Pilani, K K Birla Goa Campus, Goa, India

**Keywords:** hemoglobin-based oxygen carrier, heme stability, thermal stability, heme retention, autooxidation, cooperativity, oxygen affinity, Hb0.1 β-F41K

## Abstract

Recombinant hemoglobin-based oxygen carriers (rHBOCs) have several potential advantages as blood substitutes in transfusion medicine, especially in emergency situations. However, the wide use of rHBOCs in humans has been limited by challenges including hypertension due to nitric oxide scavenging, autoregulatory responses, and rapid heme dissociation. Among these, heme dissociation remains a critical unresolved issue, as it leads to toxicity and compromises oxygen delivery efficiency. Heme retention in the globin moiety is a key problem that needs to be solved to develop recombinant HBOCs as safe transfusion agents. Here, we report the results of protein engineering experiments to enhance heme retention and thermal stability with the aim of designing stable HBOCs. We successfully introduced a mutation into recombinant human Hb0.1 (rHb0.1), named β-F41K, that significantly reduced rates of heme dissociation and auto-oxidation while simultaneously maintaining thermal stability and oxygen affinity at levels well suited to respiratory gas transport *in vivo*. The higher rate of heme retention in recombinant Hb0.1 β-F41K makes this protein an especially promising HBOC prototype.

Globally, there is an increasing demand for a sustainable and substantial artificial blood substitute due to various factors, including the rising need for blood products, the emerging risk of new blood-borne diseases, challenges in matching rare blood types, and religious objections to blood products (1). Furthermore, there is a growing recognition that at lower doses, these substitutes can aid in oxygen delivery to compromised tissue, even when sufficient red blood cells are present. In such cases, these products do not solely provide oxygen but facilitate its transfer from red cells to hypoxic tissue. Referred to as "oxygen therapeutics," these products are proposed to offer benefits in a range of conditions, such as trauma, sickle cell disease, and subarachnoid hemorrhage (2).

Recombinant hemoglobin (rHb) shows great potential as a cell-free oxygen carrier in transfusion medicine, offering several advantages over stored blood, including extended shelf-life and reduced risks of disease transmission and harmful immune responses (3, 4, 5, 6). However, the widespread utilization of hemoglobins (Hbs) as blood substitutes in humans has been hindered by various properties of recombinant hemoglobin-based oxygen carriers (rHBOCs), which can lead to hypertension, cardiovascular complications, gastrointestinal, hepatic, pancreatic, central nervous system, renal effects, and HBOC-mediated nitric oxide (NO) scavenging and heme-associated toxicities (7, 8, 9, 10, 11, 12). Protein engineering strategies based on rational mutagenesis have addressed numerous challenges associated with rHBOCs. Nonetheless, the persistent challenge remains the high rate of rapid heme dissociation characteristic of current rHBOCs (13). Given the high toxicity of free heme, ensuring its proper incorporation and retention within the globin protein is crucial. However, protein engineering efforts to enhance heme retention face the dilemma of introducing mutations that increase heme retention without adversely affecting oxygen affinity or susceptibility to heme auto-oxidation. Such unintended alterations could compromise the oxygen-transport capabilities of the rHBOC. This study aims to address specific concerns regarding globin instability and rapid heme loss, with the goal of designing a stable HBOC suitable for emergency use as a blood substitute.

Genetically cross-linked tetramers, such as rHb0.1, were the earliest approach to reduce dissociation into dimers, a key step that facilitates hemin release (14). For instance, at low protein concentrations (∼5 μM), cross-linked rHb0.1 showed significantly lower heme dissociation rates compared to non-cross-linked rHb0.0 and HbA dimers, especially from the β-subunits, underscoring the importance of quaternary stabilization in mitigating heme loss (14). Despite the structural advantages of cross-linking, point mutations within genetically fused rHbs have revealed that the local heme environment also plays a critical role. For example, β-K82D (Providence) and β-N108K (Presbyterian) mutations increased the heme dissociation rate from β-subunits by 2- to 3-fold compared to wild-type rHb0.1, indicating that certain substitutions can override the protective effects of quaternary stabilization (15).

The cyanobacteria *Synechocystis* sp. PCC6803 hosts a remarkably stable hemoglobin (Hb) that maintains heme integrity even under extreme *in vitro* conditions. This exceptional heme stability was attributed to a covalent bond between the non-axial His117 and a heme porphyrin 2-vinyl atom (16, 17, 18, 19). Expanding on this discovery, we successfully replicated this unique stability in myoglobin (Mb) by introducing a similar covalent linkage between the heme vinyl and His107 (substituted with Ile). The engineered Mb exhibited enhanced stability without compromising its oxygen-binding and other properties. Covalent linkages involving amino acid side chains other than His (such as Lys, Glu, and Asp) to iron or methyl groups of heme have also been documented in heme proteins like *Chlamydomonas reinhardtii* Hb and myeloperoxidase, respectively (20, 21). These covalent bonds appear to significantly improve heme stability or retention of the prosthetic group within the protein matrix. Based on this rationale, we propose that similar stability could be engineered into recombinant hemoglobin (rHb) of human origin, potentially mitigating rapid heme dissociation and facilitating the development of stable HBOC prototypes. In this study, we detail our experimental endeavors to enhance the properties of one such prototype, rHb0.1, which is genetically engineered to produce substantial amounts of folded protein (22, 23). The construct includes mutations α(G15A)β(G16A/H116I) to strengthen the α1β1 interface with enhanced apoglobin stability and V1M mutations in the β chains for their expression in *E.coli*. Moreover, it is genetically cross-linked with a glycine linker between two α-polypeptides [Arg(141)-Gly-Val(1)] to prevent the dissociation of αβ dimers under normal physiological conditions, even when very dilute (23).

Rational mutagenesis may enable rHb0.1 described earlier to augment its heme stability by inserting appropriate covalent linkage or comprehensive non-covalent interactions from amino acid side chains to heme. Thus, attempts were made to introduce mutations in rHb0.1 that enhances heme retention ability. A collection of recombinant hemoglobin was created through rational, structure-based design targeting improvements in heme retention, oxidative and thermal stability with optimal oxygenation properties. Among these, β-F41K demonstrated favorable characteristics, and comparisons to other mutants in the collection were carried out to establish its unique suitability as a potential blood substitute.

Our study focused on improving the heme retention *via* a hemin-proximal point mutation β-F41K located near the heme vinyl group in the β-subunit. This residue, situated at position C7, is uniquely positioned to form new covalent bonds or electrostatic and hydrogen bonding interactions with the heme moiety, thereby stabilizing it without altering tetrameric assembly. This novel approach deviates from the traditional quaternary stabilization paradigm by directly modulating the local heme microenvironment. The mutant rHb-β-F41K demonstrated a notable decrease (∼3–4 fold) in hemin dissociation rates compared to rHb0.1 WT, along with enhanced thermal stability, exhibiting a T_m_ of approximately 62 °C. This mutation had a minimal impact on the polypeptide stability and heme coordination of the protein. Additionally, the engineered rHb mutant exhibited reduced autooxidation rate compared to the wild-type protein. Notably, rHb-β-F41K displayed significantly reduced oxygen affinity compared to rHb0.1, similar to that of red blood cell (RBC) Hb (HbA) in the presence of allosteric effector molecules such as KCl and 2,3-DPG. Such characteristics position the mutant as a potential candidate for the development of HBOCs. The initial success in engineering heme stability in rHb0.1 offers promising prospects to produce stable rHBOCs with appropriate oxygen affinity.

## Results

### Identification of amino acid side chains to be mutated in rHb0.1 using a structural analysis approach

**Figure 1:**
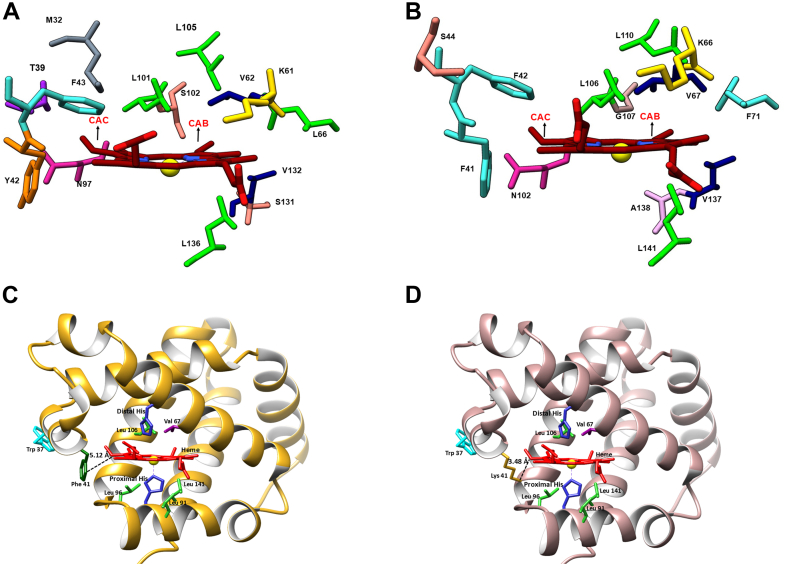
**Structural representation of recombinant human hemoglobin (PDB ID:**1C7C**).** Various key side chains decorating the heme pocket of (*A*) α-chain and (*B*) β-chain of recombinant human hemoglobin and putative amino acid side chain targets for mutation to His, Lys, Glu, and Asp within 3 Å, 4 Å, 5 Å, and 6 Å radius of heme. *C and D*, model structure of rHb0.1WT and rHb-β-F41K showing the sites of mutations in the β subunit. The ribbon drawing was generated from the coordinates for the crystal structure of the deoxy form of rHb1.1 (PDB code 1ABW). The side-chain orientations for Trp-37, Phe-41, His-63, His-92, Leu-91, Leu-96, Leu-106, and Leu-141 were taken from this structure. Distance from the heme to Phe41 is 5.12 Å and to Lys-41 is 3.48 Å. The structure was deduced using UCSF Chimera.

With the aim of engineering heme stability into rHb0.1, *in silico* analysis was performed to identify the possible α- and β-chain amino acids that could be mutated to His, Asp, Glu, and Lys to introduce an additional covalent linkage with the heme vinyl group. Using the Swiss-PDB viewer, potential residues that are in the vicinity of heme CAB and CAC (vinyl heme) were identified at different distances (in Å). The α- and β-chain sites that were in topologically equivalent positions to His117 of *Syn*Hb were identified by structural analysis (superimposition). It was seen in the crystal structure of *Syn*Hb (24) that His117 was covalently linked to the heme vinyl (CAB) atom. The heme vinyl (CAB/CAC) atom of α- and β-chains was thus targeted for the introduction of covalent modification by His and Lys, to be introduced in a suitable location in rHb. In addition, the crystal structure of myeloperoxidase showed that methylated pyrrole rings I and III are covalently linked *via* ester bonds to Asp109 and Glu258 (20). Thus, the four-heme methyl (CMA/CMB/CMC/CMD) atoms of α- and β-chains were targeted for potential covalent modification by Asp and Glu, to be introduced in a suitable location in rHb. Using the Swiss-PDB viewer, potential residues that are in the vicinity of heme vinyl (CAB/CAC) and four heme methyl (CMA/CMB/CMC/CMD) atoms were identified at different distances (in Å) (25). Up to 3 Å, no residues were found in the vicinity of heme vinyl and methyl groups. The residues obtained at a distance of 4 Å to 6 Å were targeted for mutations. No attempts were made beyond 6 Å since the desired mutated residues may not come in close contact with the vinyl or methyl groups at higher atomic distances. Out of these residues, Phe 98 and Val 93 in the α-subunit and Phe 103 and Val 98 in the β-subunit were not mutated as they were shown to be involved in “waterproofing” the heme pocket (26). Target residues were mutated to His, Lys, Glu, and Asp *in silico* using the crystal structure of rHb1.1 (PDB ID: 1C7C). Thus, the potential target residues up to 6 Å distance from the heme vinyl carbon were selected for mutation and are shown in Figure 1, *A* and *B*. Additionally, it was noted that Phe resides approximately 5.12 Å away from the heme vinyl carbon atom. Upon mutation to Lys, this distance decreased to approximately 3.48 Å, as illustrated in Figure 1, *C* and *D*. A summary of target mutants is listed in Table S1, *A* and *B*.

### Mutagenesis, expression, purification, and spectroscopic characterization of recombinant human hemoglobin (rHb0.1) mutant proteins

Using rationale-based comparative mutagenesis, as described above, a large number of mutants were generated as summarized in Table S1*A*. Single mutations may or may not induce the desired heme retention ability in rHb since α- and β-chains have their individual characteristics with regard to stabilities. Hence, a combination of mutants was also generated where either two mutations were introduced in the same chain (either α- or β-) or appropriate mutations were introduced in both chains. The list of such mutations is shown in Table S1*B*. After generating mutants, attempts were made to express these in *Escherichia coli*. Most of the mutants were soluble and hence were purified easily. Some mutants showed low expression yields and could not be purified on a large scale and hence were not characterized further. The recombinant human hemoglobin wild-type (rHb0.1) and its mutant proteins were expressed as described in the Methods section.

**Figure 2:**
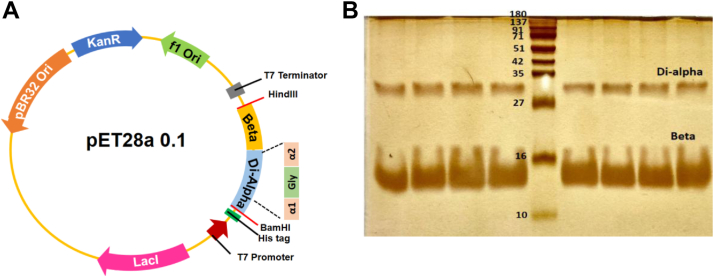
**Expression and purification of recombinant human hemoglobin.***A*, expression vector pET28a was used for the expression of rHb gene cloned between BamHI and HindIII restriction sites. His 6X tag (HHHHHH) is present at the N-terminus of di-alpha gene aiding in purification using Ni-NTA affinity chromatography. *B*, silver stained SDS-PAGE of purified recombinant human hemoglobin wild type protein. The purification was carried out by using various chromatographic techniques and analyzed by electrophoresis on 15% SDS-polyacrylamide gel. Lane 5 molecular weight marker. The rest of the lanes are for the different fractions of the protein eluted after gel filtration chromatography.

The 6X-His (at N-terminal) tagged rHb0.1 WT protein and all its mutants were expressed under the control of T7 promoter (Fig. 2*A*). The expression product has one di-α glycine-linked and two β subunits with V1M mutation in both chains. rHb0.1WT also contains stability mutations di-α(G15A)β(G15A/H116I), which are responsible for increased apoglobin stability and its expression in *E.coli* (13). The protein was purified using Ni-NTA affinity chromatography and gel filtration chromatography. The purified bright red protein was subjected to SDS-PAGE as shown in Figure 2*B*. The gel indicated that the proteins were purified to homogeneity, since bands specific for only α- and β-subunits were observed. The purified rHb0.1 WT protein migrated with an apparent molecular mass of ∼16 kDa for the β-chain and ∼32 kDa for the di-α-chain on SDS-PAGE, values consistent with those deduced from the amino acid sequence.

**Figure 3:**
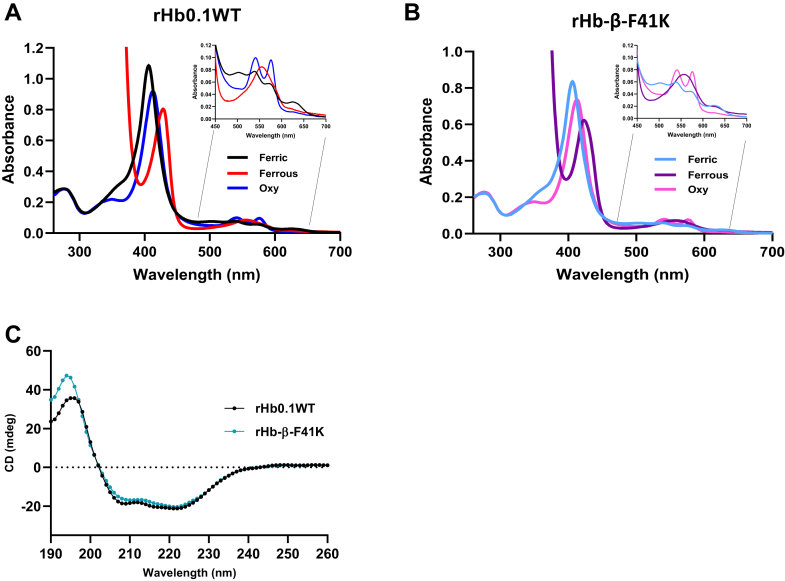
**Spectral characterization of wild type and mutant protein.***A* and *B*, absorbance spectra of the purified protein rHb0.1WT and rHb-β-F41K, respectively, in the ferric, ferrous and oxygen-bound forms. The inset figure shows an enlarged view of the absorbance spectra in the range of 500 to 700 nm, highlighting the Q-band region. *C*, secondary structure of recombinant human hemoglobin wild type (*black*), and rHb-β-F41K (*blue*) was monitored by far UV-CD spectroscopy in the range of 260-190 nm. The identical spectra indicate that the mutation did not perturb the overall conformation of the protein.

rHb0.1WT and all the mutant proteins were characterized using absorbance, fluorescence, and CD spectroscopy at a protein concentration of 0.1 mg/ml at pH 7.4 to identify significant differences in polypeptide conformation and ligand binding, if any. The unique absorbance spectra of Hbs in the UV-visible range provide valuable insights into their oxidative states, the formation of ligand complexes, iron complexes, and the structural stability of these proteins. (27). The absorbance spectra of the recombinant hemoglobin in reduced (Fe^+2^), oxidized (Fe^+3^), and ligand-bound states are shown as representative spectra (Fig. 3, *A* and *B*). The absorbance spectra of rHb0.1WT and the representative mutant β-F41K mutant proteins displayed Soret wavelength maxima and Q-bands typical of Hbs and are summarized in Table 1. The absorbance spectra of rHb0.1WT and mutant β-F41K proteins showed Soret peaks at 406 nm (Fig. 3, *A* and *B*). The Q-bands for both proteins were also similar. The identical Soret and charge-transfer spectra indicate that the introduction of Lys at specified positions in rHb0.1 did not alter the heme polypeptide interaction in the heme pocket. The majority of the other mutants investigated displayed similar results (data not shown).

**Table 1:** Summary of the Soret peak wavelength and Q-bands of rHb0.1 WT and representative mutant protein in various oxidation and ligand bound states

Protein	Ferric		Ferrous		Fe-O_2_	
	Soret (nm)	Q-band (nm)	Soret (nm)	Q-band (nm)	Soret (nm)	Q-band (nm)
rHb0.1 WT	406	507	428	556	414	540, 576
rHb-β-F41K	406	505	429	557	412	541, 574

CD spectral profiles (Fig. 3*C*) showed that rHb0.1WT and its mutant β-F41K are α-helical proteins with double negative minima at 208 nm and 222 nm. The mutant had similar ellipticities as wild-type protein, indicating that both proteins had similar fold and secondary structural arrangements. Similar analyses were performed for all the other mutant proteins, and no major changes were noted (data not shown).

### Qualitative heme extraction assay indicated that both recombinant human hemoglobin wild type (rHb0.1) and mutant proteins dissociate heme, typical of hemoglobins

**Figure 4:**
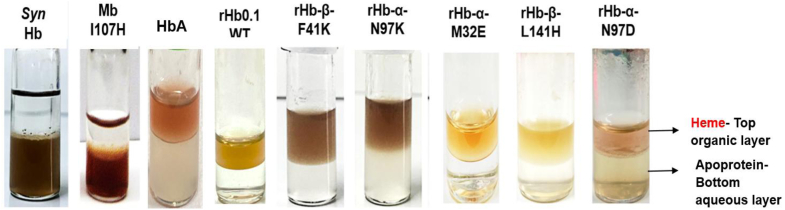
**Comparison of the heme retention abilities of recombinant human hemoglobin wild type and representative mutant proteins.***Syn*Hb WT and MbI107H (negative controls) do not release heme in the organic layer, but HbA, rHb0.1 (WT), rHb-β-F41K, rHb-α-N97K, rHb-α-M32E, rHb-β-L141H, and rHb-α-N97D release heme in the organic layer (*top*).

The aim for creating mutants as above was to ensure that the rHb protein gained the ability to retain heme within the protein matrix, even under conditions of extremes of pH and in the presence of denaturants. The heme retention ability was evaluated for the mutants in comparison to rHb0.1 WT using a standard assay. Heme extraction assay performed at low pH showed that rHb0.1 wild-type and all its mutants lost heme to the organic layer typical of globins, whereas *Syn*Hb and MbI107H protein were able to retain heme in the aqueous layer within the protein (Fig. 4). This indicated that the mutations in the heme pockets of α- and β-chains of rHb0.1 were unable to introduce covalent linkage with the heme group, thus leading to the release of heme from the protein matrix (Fig. 4). This method presents a viable and economically efficient approach to ascertain the presence or absence of covalent linkages.

### Kinetic measurements indicated that the heme dissociation rates of rHb-β-F41K mutant are significantly reduced

Qualitative assessment of the heme stabilities of rHb0.1 and the mutant proteins (Fig. 4) showed no enhancement in heme affinity as indicated by the heme extraction assay (Qualitative heme extraction assay indicated that both recombinant human hemoglobin wild type (rHb0.1) and mutant proteins dissociate heme typical of hemoglobins). Hence, it was necessary to probe the heme affinities using a quantitative assay that measures the rate of heme dissociation from the globins. To evaluate heme transfer rates, we first measured the absorbance alterations upon the binding of the acceptor molecule, ApoMb, with heme released from the ferric form of Hb, resulting in the formation of holoMb. ApoMb displays distinctive spectral characteristics as it effectively extracts heme from Hb (and Mb), leading to significant absorbance changes conducive to reactions at low heme concentrations. These changes encompass a reduction in intensity, a slight red shift of the Soret maximum, and the emergence of a prominent band at 600 nm, imparting a green color. The heightened absorption at 600 nm is attributed to the direct coordination of the tyrosine phenol side chain with the iron atom. Heme in Hb in ferrous (Fe^2+^) state is covalently attached to proximal His, which prevents its release under normal physiological conditions. Auto-oxidation facilitates the release of heme from the protein matrix due to the considerable weakening of this bond and leads to the formation of the ferric (Fe^3+^) form of the proteins. Heme retention inside the protein matrix is influenced by different numbers and types of interactions between heme and protein. Thus, the kinetics of heme dissociation provides a better understanding of Hb stability *in vitro*, which may be reflective of function as well. Here, the effect of rationale-based mutagenesis in the heme pockets on the retention of heme was quantified by a rapid and convenient assay as described earlier (28).

**Figure 5:**
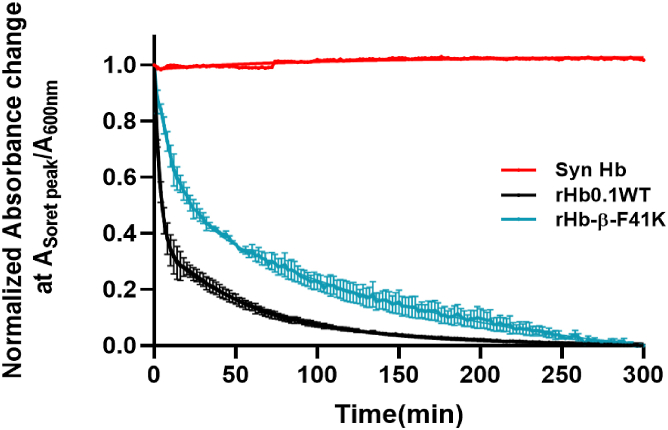
**Time courses for heme dissociation from SynHb (*red*), rHb0.1 WT (*black*) and rHb-β-F41K mutant in 0.1 M phosphate buffer at pH 7.4, 37 °C.** The "*brown*" metHb at a concentration of 3 μM was mixed with 30 μM H64Y/V68F apoMb, which transitions to "*green*" upon hemin scavenging. With a 10-fold excess of the scavenger, the observed rates reflect hemin dissociation from the Hb sample. Notably, the rate of hemin dissociation from the mutant α- and β-subunits in rHb-β-F41K is approximately 3- and 4-fold slower, respectively, compared to those from rHb0.1 wild-type α- and β-subunits. Changes in absorbance ratio (A_Soret peak_/A_600nm_) of the globins were normalized and plotted against time. The traces represent the average of three experiments with the standard deviations for each point shown as error bars. The lines represent fits to two exponential expressions with the fast phase representing hemin dissociation from β-subunits and the slow phase, dissociation from α-subunits.

The results of heme dissociation assays are shown in Figure 5. *Syn*Hb was used as a negative control since this stable globin does not dissociate heme at all under the conditions of the experiment. Biphasic kinetics of hemin loss were observed in recombinant human hemoglobin, attributed to the presence of two distinct subunits (α and β). The rapid phase corresponds to hemin loss from ferric β-subunits, while the slower phase denotes hemin loss from α-subunits (14). According to Hargrove *et al.*, isolated alpha subunits of HbA exhibit a slower heme dissociation rate (12 h^−1^) compared to isolated beta subunits (40 h^−1^). Furthermore, even within dimers and tetramers, the alpha subunits consistently show lower heme dissociation rates (0.6 h^−1^ and 0.3 h^−1^, respectively) than the beta subunits (15 h^−1^ and 1.5 h^−1^, respectively). Native hemoglobin dimerizes into equivalent α1β1 dimers in dilute solutions (14). rHb0.1WT demonstrates enhanced dissociation dynamics, featuring rate constants of 0.96 h^−1^ for the α-chain and 16.2 h^−1^ for the β-chain. The various mutants exhibited a diverse range of heme dissociation rate constants, some being similar to rHb0.1 WT while others having much faster heme dissociation rates. Fig. S1 shows the percentage of heme dissociation from mutants with slow heme release as compared to rHb0.1 WT. None of the mutants exhibited rates of heme retention to the same extent as *Syn*Hb. The heme dissociation rates of the globins investigated here are summarized in Table S2. It was evident that only rHb-β-F41K exhibited a 3- to 4-fold increase in heme retention as compared to the wild type (Table S2). Lys with its long side chain might have interacted with heme moiety, thereby preventing faster dissociation. Nonetheless, the interaction of Lys with heme is not covalent in nature like His117 *Syn*Hb or His107 in the stable Mb mutant (24, 29). The covalent linkages in *Syn*Hb and the Mb mutant do not allow heme to dissociate (Fig. 5), while heme is eventually lost in this rHb mutant. The order of heme dissociation rates is: rHb0.1 WT >>> ≅ rHb-β-F41 K >> *Syn*Hb. While there are no universally accepted quantitative thresholds for each parameter, comparative values from native HbA and prior HBOCs serve as reasonable benchmarks. For example, Meng *et al.* (30), reported heme dissociation rates from HbA, which we used to contextualize the improved heme affinity seen in β-F41K (∼2.6-fold better than HbA in the β-subunit and ∼2.5-fold in the α-subunit). This improvement is substantial and represents a meaningful step toward enhanced stability of HBOCs.

In summary, we used a rational, mutagenesis-based protein engineering approach to increase heme affinity in recombinant human hemoglobin (rHb0.1), while avoiding deleterious side effects to other biochemical properties. This finding brings us one step closer to the development of safe and stable rHBOCs that have ligand-binding properties optimized for circulatory oxygen transport with possible application of rHb0.1-β- F41K as a HBOC prototype.

### Recombinant hemoglobin 0.1 wild type is a tetrameric globin

**Figure 6:**
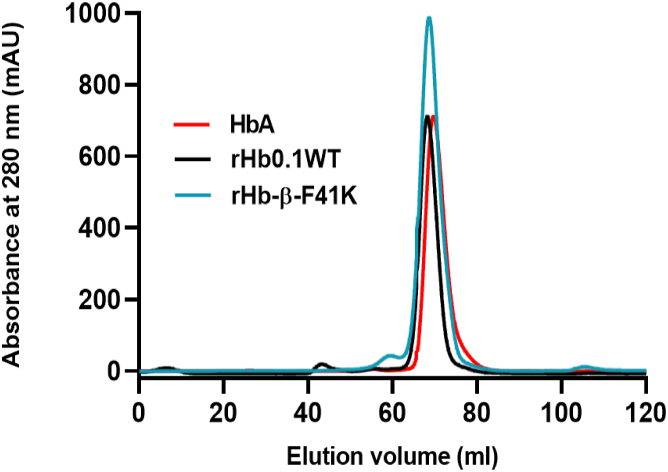
**Superdex 75 10/300 Gl chromatogram profiles.** rHb0.1 WT and rHb-β-F41K exhibits the tetrameric quaternary structure as control protein native HbA. Native HbA was used as a standard protein whose molecular weight is known to be ∼ 64 kDa.

To determine the oligomeric state of rHb protein in solution, size exclusion chromatography was performed. Protein of concentration 15 to 20 mg/ml (∼300 μM heme) was subjected to FPLC along with control protein HbA at a flow rate of 1 ml/min. Control protein HbA was used to obtain the reference for the molecular weight *versus* elution volume. Notably, both rHb0.1 WT and heme-stable mutant rHb-β-F41K exhibited a peak with an elution volume identical to that of a native HbA. Upon chromatogram analysis, it was evident that both the rHb0.1 WT and its mutant rHb-β-F41K formed tetramer (α_1_α_2_β_1_β_2_) as denoted by their elution volume of approximately 70 ml (Fig. 6).

### Heme stabilized mutant rHb-β-F41K maintains tetrameric structure like other rHb mutants with fast heme dissociation kinetics

**Figure 7:**
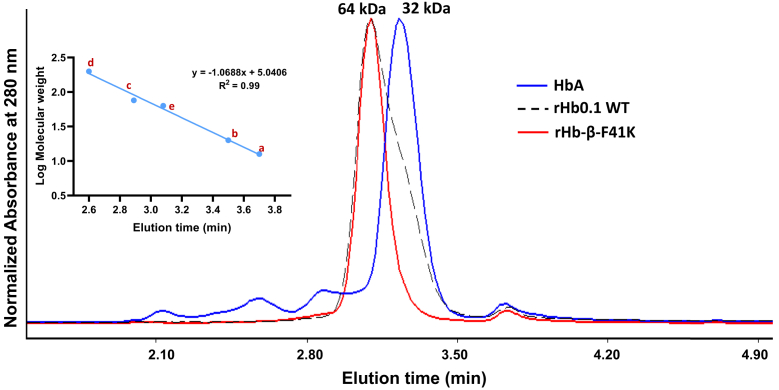
**Determination of the quaternary structure of recombinant hemoglobin β-F41K mutant in solution**. HPLC analysis of rHb proteins on a size exclusion column at protein concentrations of 3 μM (e), with a, b, c, d and e depicting standard proteins (Lysozyme: 14.3 kDa, Trypsin: 24 kDa, Conalbumin: 76 kDa, Catalase: 240 kDa, and rHb0.1WT: 64 kDa, respectively) of known molecular weights. HbA showed dissociation of tetramer, unlike rHb0.1WT and its heme stabilized mutant β-F41K at the minimal concentration used for investigating heme dissociation kinetics.

The increased rates of hemin loss observed in some Hb mutants, as listed in Table S2, are relative to rHb0.1WT and may result from their enhanced tendency to dissociate into dimers, which are known to lose hemin approximately 10 times faster than tetramers (31). To evaluate their tetramer-dimer dissociation propensity at a concentration of 3 μM, utilized for heme loss kinetics, in comparison to the heme-stabilized mutant rHb-β-F41K, we employed analytical gel filtration analysis. The proteins at a concentration of 3 μM were subjected to chromatography alongside marker proteins at a flow rate of 1 ml/min. Various marker proteins including Catalase, Conalbumin, Trypsin, Lysozyme, and rHb0.1WT (at 300 μM) with molecular weights of 240 kDa, 76 kDa, 24 kDa, 14.3 kDa, and 64 kDa, respectively, were utilized to generate a reference plot for molecular weights *versus* elution time (inset of Fig. 7). rHb0.1WT and its various mutants like rHb-β-F71H, rHb-α-N97K, rHb-α-Y42H, rHb-β-S44H/β-N102K, rHb-α-L66H/α-N97K, and rHb-β-S44H/α-N97HH eluted at approximately 3.06 min, corresponding to a 3.06 ml elution volume, similar to the heme-stabilized mutant rHb-β-F41K (Fig. S7). HbA was used as a control protein which eluted at 3.2 min indicating that it dissociates from the tetramer at very dilute concentrations used in this study. Based on standard curve analysis, the molecular weight of these mutants was approximately 64 kDa, indicative of a tetrameric state of the protein at the minimal concentration utilized for heme dissociation kinetics (Fig. 7). Thus, the rapid heme dissociation kinetics from these mutants is not due to their tendency to dissociate into dimers and therefore maintains the tetrameric structure like our heme-stabilized mutant rHb-β-F41K.

### Effect of pH on the structural stability and heme retention of recombinant hemoglobins

The native conformation of a protein, which is crucial for its biological activity, is stabilized by various forces (covalent and non-covalent). The perturbation of these forces can cause denaturation, leading to a loss of structure and activity. Since a protein's activity is inherently dependent on its three-dimensional structure, even subtle changes in conformation can significantly affect its functional properties, including binding affinity, stability, and catalytic or transport efficiency (32). Analyzing how recombinant human hemoglobin (Hb) responds to stressors like temperature, pH, and denaturants (*e.g.*, guanidinium hydrochloride) provides insights into its conformational stability. Since hemoglobin’s function depends on its structural integrity for oxygen transport, denaturation studies reveal its robustness under physiological and stress conditions. The relationship between heme binding and protein function is key, as heme release causes loss of structure and activity (33).

The impact of pH on the spectral characteristics of recombinant hemoglobin (Hb) was examined, recognizing pH as a critical factor influencing protein stability. Amino acids comprising proteins exhibit ionizable properties, and alterations in pH induce changes in amino acid ionization, subsequently influencing protein stability through the disruption of non-covalent interactions. Protein unfolding is commonly observed at extreme pH values, leading to the destabilization of Hbs. Consequently, the Soret peak experiences a blue shift in absorbance wavelength accompanied by a concurrent reduction in intensity (27). Notably, a progressive reduction in the Soret peak intensity of both rHb0.1WT and rHb-β-F41K was observed under both acidic and alkaline conditions, and HbA was used as a control protein in this study. The alterations in the Soret wavelength maxima of rHb0.1 WT and its mutants were monitored in response to changes in pH, in the range of 2.0 to 11.0. At pH 3, rHb-β-F41K retains the heme moiety when compared to native HbA and rHb0.1 WT, indicating higher stability. The complete spectra of the proteins at pH 7.0 and pH 2.0 revealed that rHb-β-F41K displayed a characteristic Soret peak (366 nm) at pH 2.0, which is similar to free heme at the same pH(Fig. S4, *A*–*C*).

### β-F41K replacement enhances thermal stability of rHb0.1 WT

**Figure 8:**
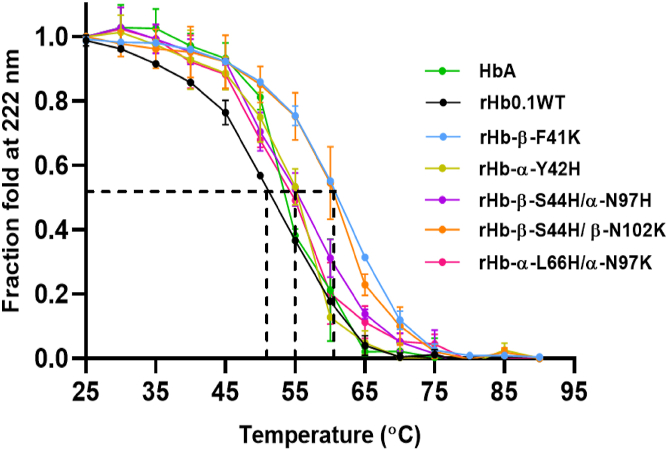
**Changes in the secondary structure of proteins were monitored with the increase in temperature using CD spectroscopy.** CD_222 nm_ values were plotted against temperature in the range of 25-90 °C. rHb-β-F41K and rHb-β-S44H/β-N102K are thermally stable mutants compared to rHb0.1WT and other mutants with T_m_ at around 62 °C. The data is presented as an average of three independent experiments, with the standard deviations for each point shown as error bars.

The engineered mutant rHb-β-F41K displayed structural characteristics similar to wt rHb0.1 and had higher heme affinity and stability to chemical perturbation. To be useful as HBOCs, the protein should also have polypeptide stability similar to rHb0.1 or better. Three-dimensional structures of proteins exist in the functional form only within the limits of specific conditions inside the cellular environment. The offset of these conditions leads to the denaturation and unfolding of protein structure. Temperature is an important factor that affects protein stability. The effect of temperature on protein stability was investigated for rHb0.1WT, its α-chain mutants, β-chain mutants, and its heme-stable mutant rHb-β-F41K using circular dichroism spectroscopy. The unfolding curves provide global insight into the thermal denaturation of proteins and help in the suitable calculation of T_m_ (transition thermal mid-point), a quantitative indicator of secondary structural stability and conformational changes. It was found that while rHb0.1WT exhibited a T_m_ of ∼52 °C, β-F41K showed a T_m_ of ∼62 °C, which is significantly higher as compared to rHb0.1 WT (Fig. 8). Thermal stability experiments thus indicated that the mutation did not influence the stability of the globin (β-F41K) and thus no loss of tertiary structure of proteins is expected for this mutant. This mutant (β-F41K) thus exhibited a stable apoprotein and higher heme affinity, thus indicating its utility in the production of HBOCs. The other mutants displayed a wide range of T_m_. For instance, mutants like α-Y42H, α-L101E, α-V132K, β-N102E, α-L66H/α-N97K, and β-S44H/α-N97H showed T_m_ in the range of 50 to 55 °C, as shown in Table 2. Moreover, the thermal stability of β-F41K was significantly higher than both HbA and rHb0.1WT, suggesting it will be more amenable to long-term storage and use in extreme conditions, an essential trait for real-world HBOC applications. While the T_m_ of 62 °C is not a gold standard, it exceeds the stability of unmodified Hb (HbA and rHb0.1WT), indicating progress toward a robust prototype.

**Table 2:** Comparison of melting temperature (apparent T_m_) of rHb0.1 wild type and mutant proteins measured using circular dichroism spectroscopy

Protein	Apparent T_m_ (°C)
rHb0.1 WT	52 ± 0.007
HbA	54 ± 0.030
rHb-β-F41K	62 ± 0.005
rHb-α-Y42H	55 ± 0.057
rHb-α-L101E	50 ± 0.035
rHb-α-V132K	55 ± 0.062
rHb-β-N102E	52 ± 0.021
rHb-α-L66H/α-N97K	55 ± 0.003
rHb-β-S44H/β-N102K	62 ± 0.112
rHb-β-S44H/α-N97H	55 ± 0.051

### rHb-β-F41K showed similar secondary structure in the presence of guanidium hydrochloride but higher stability to pH compared to rHb0.1WT

**Figure 9:**
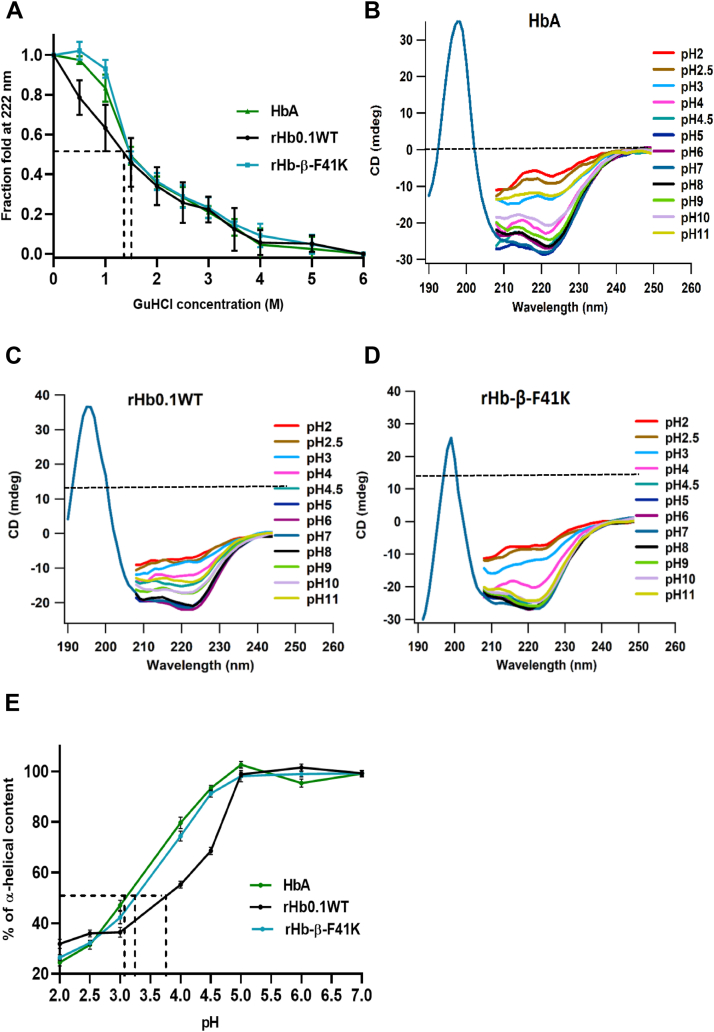
**GuHCl and pH stability studies of native HbA (Control), recombinant hemoglobin 0.1 wild type and mutant protein β-F41K**. *A*, changes in the secondary structure were monitored with increase in the GuHCl concentration using far UV-CD signal at 222 nm; the data shows similar alpha-helical stability for both proteins as HbA (control protein). Three independent experiments were performed for this study. *B–D*, far-UV CD spectra of HbA, rHb0.1WT and β-F41K, respectively, incubated at pH range from 2.0 to 11.0 to assess the changes in secondary structure. The rHb0.1WT is stable from pH range 5.0 to 8.0 whereas the mutant exhibits better stability in the pH range 4.5 to 11.0. *E*, % α-helical content was calculated at CD_222nm_ (mdeg) and were plotted as a function of pH from 2.0 to 7.0. Spectra were recorded at a protein concentration 0.15 mg/ml. rHb0.1 WT and mutant protein displayed maximum ellipticity value at pH 7.0. Three independent experiments were performed for this study.

To determine whether the β-F41K mutation altered the stability of the helical structure compared to HbA and rHb0.1WT, we specifically monitored changes in secondary structure using circular dichroism (CD) at 222 nm, which is sensitive to α-helical content. Since GuHCl is a well-established chaotropic agent known to disrupt hydrogen bonds and destabilize protein structures, it provides a quantitative means of comparing protein stability based on resistance to denaturation (34). Our results show that β-F41K exhibits similar secondary structure stability, with apparent denaturation midpoints at ∼1.4 M GuHCl for HbA and ∼1.3 M for rHb0.1WT (Fig. 9*A*). This indicates that the mutation does not significantly disrupt the protein’s stability under chemical denaturation conditions. The changes in the secondary structural content of rHb-β-F41K in comparison to native HbA and rHb0.1WT at variable pH was investigated by CD (Fig. 9, *B*–*D*). Again, a steady decrease in α-helical content was observed for the proteins in pH extremes. Unlike native HbA and rHb0.1WT, the mutant retained the characteristic α-helical spectrum even at pH 4.5, suggesting a stable core of the secondary structure. The pH-induced alterations in terms of a gradual decrease in α-helicity of both the proteins were comparable till pH 5 but at pH 4.5 rHb0.1WT lost almost 30.42% of α-helicity whereas β-F41K lost only 7.73% comparable to native HbA, which lost only 5.92% of α-helicity. In other words, our heme-stabilized mutant was able to retain 50% of α-helicity at ∼ pH 3.25 similar to native HbA. (Fig. 9*E*). Moreover, the mutant demonstrated significant stability even in the alkaline pH range from 8.0 to 11.0 without changes in its secondary structure (Fig. 9*D*). Overall, the pH stability experiments suggested that β-F41K was more resistant to pH-induced changes in secondary structure than rHb0.1WT.

### The heme-stable mutant rHb-β-F41K reveals slower autooxidation kinetics compared to the wild-type protein (rHb0.1)

**Figure 10:**
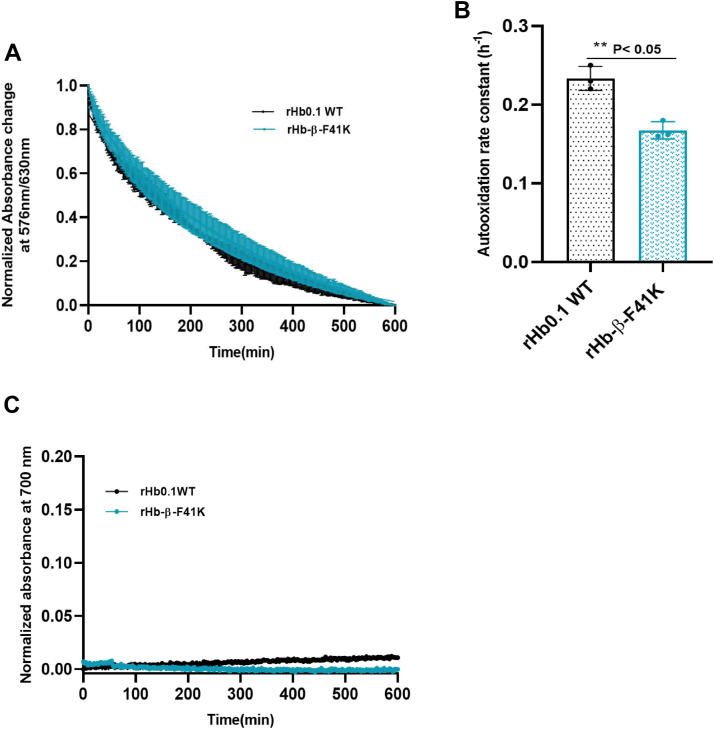
**Influence of mutations on auto-oxidation in hemoglobin.***A*, normalized absorbance time courses for the autooxidation of recombinant human hemoglobins at 37 °C in air-equilibrated 50 mM potassium phosphate, 1 mM EDTA, pH 7.5, containing 3 mmol/mol heme each of catalase and superoxide dismutase. The data is presented as an average of three independent experiments with error bars. *B*, bar graphs representing the average of autooxidation rate (h^−1^) of both rHb0.1WT and rHb-β-F41K. Each bar represents mean ± S.D. of three experimental repeats. Statistical differences between rHb0.1WT and rHb-β-F41K were analyzed using an unpaired *t* test, which showed significance with ∗∗*p* < 0.05. *C*, absorbance changes at 700 nm captured when 50 μM oxy-bound rHb0.1 WT and rHb-β-F41K undergo oxidation during incubation in 50 mM potassium phosphate buffer, pH 7.4, at 37 °C for 10 h.

Hb tetramer regulates oxygen affinity as well as maintains its heme-iron in a ferrous (Fe^2+^) state, with the help of enzymatic reduction systems present in red blood cells. Hb in solution autoxidizes very rapidly into a physiologically inactive ferric (Fe^3+^) state. Autooxidation of Hb leads to the unfolding and precipitation of protein as well as the release of toxic heme from the protein matrix, which causes oxidative stress (35). Hence, it was important to investigate the effect of the mutations studied here on the rate of autooxidation. Stable HBOCs must have auto-oxidation rates lower or at least similar to rHb0.1 WT protein. Autooxidation rates were measured by monitoring the absorbance changes at 576 nm and 630 nm, as described previously both at neutral pH and standard temperature and under physiology mimicking conditions (36). An estimate of the rate of autooxidation for rHb0.1 WT and its mutant were obtained by plotting the change in the ratio A576 nm/A630 nm (∗[HbO2]/[metHb]) as a function of time. The rates of auto-oxidation were calculated to be 0.16 ± 0.01 h^−1^ for rHb-β-F41K and 0.22 ± 0.007 h^−1^ for rHb0.1 WT (Table 3). These results indicate that rHb-β-F41K exhibits a slightly slower auto-oxidation rate compared to the wild-type protein under physiological conditions, consistent with its potentially improved stability (Fig. 10, *A* and *B*). We also did not observe any denaturation for the rHb0.1WT and rHb-β-F41K when autooxidation kinetics was performed at 37 °C for 10 h. Normalized absorbance changes at 700 nm were plotted over time, and no significant change in absorbance was observed for either the wild-type (WT) or the mutant. This indicates that under the experimental conditions, there was no denaturation or precipitation occurring in either sample. The lack of absorbance changes at 700 nm, a wavelength commonly associated with light scattering due to aggregation or precipitation, suggests that both the WT and the β-F41K remained soluble and structurally stable during the experiment (Fig. 10*C*).

**Table 3:** The autooxidation rates of rHb0.1 WT and its mutant proteins

S.no.	Protein	Autooxidation rate (h^−1^)
1.	rHb0.1 WT	0.22
2.	rHb-β-F41K	0.16
3.	rHb-α-Y42H	0.30
4.	rHb-α-L101E	0.46
5.	rHb-β-N102E	0.48
6.	rHb-α-V132K	0.50
7.	rHb-β-S44H/α-N97H	0.30
8.	rHb-α-L66H/α-N97K	0.48
9.	rHb-β-S44H/β-N102K	0.38

While rates of auto-oxidation were measured for several mutants investigated here (data not shown), emphasis was on the mutants which demonstrated higher heme affinities since they are the ones that are expected to have utility in the generation of heme-stable HBOCs. Thus, autooxidation data for heme stable mutants other than rHb-β-F41K was included in Fig. S2. These experiments indicated that the engineered mutation decreased the autooxidation rate of the Hb and thus is viable for the creation of HBOCs. However, the autoxidation rate of β-F41K, while lower than rHb0.1WT, is still ∼3-fold higher than HbA (k_auto = 0.043 h^−1^) (30). This is in line with previous findings that structural modifications, especially those altering oxygen affinity or pocket accessibility, often accelerate autoxidation (37). The rapid autoxidation of the HBOCs appears to be due to conformational constraints that make their heme pockets more open to the aqueous environment. These rates are not yet ideal and warrant further engineering to bring them closer to native HbA levels.

### Hydrogen peroxide-mediated oxidation revealed oxidative stability of β-F41K similar to wild type

**Figure 11:**
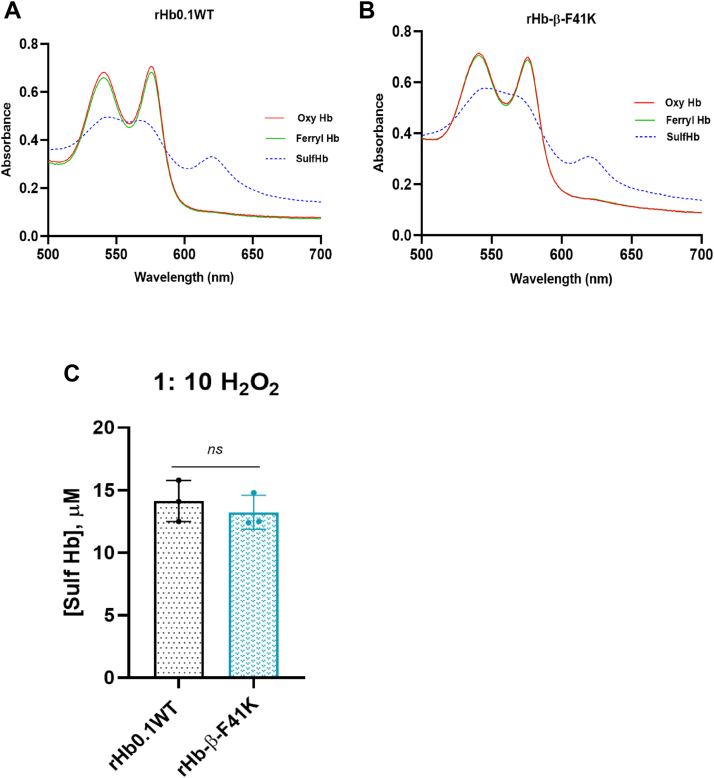
**H_2_O_2_-mediated oxidation of Hbs**. *A* and *B*, absorbance spectra of rHb0.1WT and rHb-β-F41K, respectively, obtained upon treatment of ferrous rHb with 10 equivalents of H_2_O_2_. The *red solid line* is for ferrous rHb0.1WT and rHb-β-F41K, respectively, before H_2_O_2_ treatment. The *green* solid line is for ferryl Hb formed after the addition of 10 equivalents of H_2_O_2_. SulfHb spectrum was obtained immediately after the addition of sodium sulfide (Na_2_S) (2 mM) to oxidized rHb solutions that exhibit a characteristic strong peak at 620 nm. *C*, amounts of sulfHb formed after treatment with 1:10 ratio of heme: H_2_O_2_ is calculated using extinction coefficient, Ɛ_620nm_ = 20.8 mM^−1^. Each bar represents mean ± S.D. of three experimental repeats. Statistical differences between rHb0.1WT and rHb-β-F41K were analyzed using an unpaired *t* test, which showed no significant difference (*p* > 0.05, not significant, ns).

Reactions of the rHbs with H_2_O_2_ were investigated to compare their susceptibility to chemically-induced oxidation and subsequent oxidative changes. A series of reactions were carried out between these rHbs with 10 equivalents of H_2_O_2_. Typical spectral changes in the reaction of rHbs with 1:10 ratio of heme: H_2_O_2_ are presented in Figure 11, *A* and *B*. The initial spectrum at time zero is that of oxyHb with characteristic absorption maxima at 576 nm and 541 nm, respectively. The second spectrum is that of the ferrylHb formed after the incubation of ferrous Hb with H_2_O_2_ for 10 min. The ferrylHb concentration was estimated using recently calculated extinction coefficients or derivatized to sulfHb (absorption maxima at 620 nm) by the addition of Na_2_S before it relapses back to ferric Hb *via* the autoreduction process. The results showed that both wild type and β-F41K formed similar amount of sulfHb at 1:10 ratio of heme: H_2_O_2_ (Fig. 11*C*).

### β-F41K exhibited oxygen affinity similar to RBC Hb while that of rHb-α-N97K was significantly higher than both RBC Hb and rHb0.1

**Figure 12:**
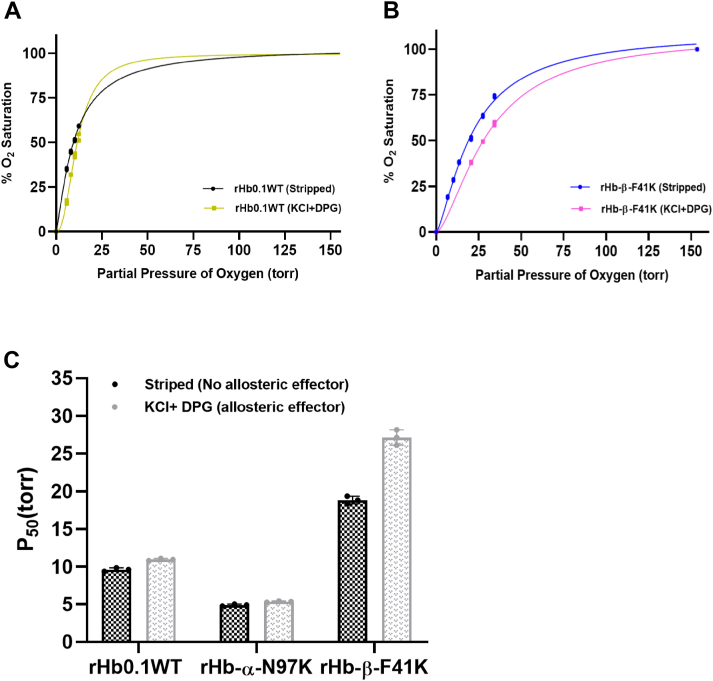
**Oxygen equilibrium curves (OECs) for recombinant human hemoglobin.***A*, wild type rHb0.1 WT, and (*B*) rHb-β-F41K mutant in absence of allosteric effector (stripped) and presence of allosteric effector (KCl and DPG), using a blood oxygen binding system. *C*, bar graph representing the average of P_50_ values of rHb0.1WT, rHb-α-N97K and rHb-β-F41K. Each bar represents mean ± S.D. of three experimental repeats.

The functionality of Hbs as O_2_ transporter or storage agent depends on the rates of O_2_ association and dissociation, which dictates their oxygen affinities. Recombinant human Hb displays high oxygen affinity compared to RBC Hb (native) due to the loss of allosteric effector molecules (which are present inside RBCs), which makes it difficult to deliver oxygen to tissues. The oxygen affinity (P_50_) of rHb is 10 mmHg, while that of RBC Hb is 27 mmHg. HBOCs should display P_50_ in the same range as RBC Hbs to be clinically relevant. Thus, the oxygen-binding parameters of recombinant human hemoglobin (wild type) and the successfully engineered stable mutant protein (β- F41K) was measured on a Blood Oxygen Binding System (Loligo Systems) (Fig. 12, *A* and *B*). One of the mutants that did not exhibit heme stability, rHb-α-N97K, was also investigated as a control protein not expected to demonstrate improved oxygen affinity. Although some mutants exhibited enhanced heme retention (as shown in Fig. S1), they were not pursued further due to limitations such as poor expression levels and reduced thermal stability. Heme stable rHb-β-F41K exhibited a significantly lower oxygen affinity (P_50_ = 27.14 ± 0.73 torr) as compared to that of rHb0.1 WT (P_50_ = 10.95 ± 0.10 torr), but a similar oxygen affinity as RBC hemoglobin (HbA) (P_50_ = 27.0 torr) in presence of allosteric effector molecules like KCl^−^ and 2,3-DPG, making it a potential candidate for the production of HBOC (Fig. 12). On the contrary, rHb-α-N97K, the control Hb^,^ exhibited oxygen affinity (P_50_ = 5.35 ± 0.06 torr) even higher than rHb0.1 WT protein, indicating that this mutant may not be suitable as an HBOC (Fig. 12*C* and Table 4). Thus, the mutant rHb-β-F41K showed higher heme retention ability, reduced auto-oxidation rate and higher polypeptide stability compared to rHb0.1 WT, characteristics favorable for HBOC. The mutated variant rHb-β-F41K exhibited 2 to 3 folds lower oxygen affinity as compared to recombinant wild-type Hb of the order of native Hb, thus emphasizing its claim as a potential HBOC prototype.

**Table 4:** P_50_ and the Hill coefficient (n_50_) of rHb0.1 WT and mutant proteins in absence and presence of allosteric effector

Protein	Stripped		KCl^−^ + DPG	
	P_50_	n_50_	P_50_	n_50_
HbA	20.0	3.0	26.3	2.7
rHb0.1	9.6 ± 0.16	1.21 ± 0.03	10.95 ± 0.10	1.91 ± 0.03
rHb-α- N97K	4.9 ± 0.09	1.41 ± 0.06	5.35 ± 0.06	1.44 ± 0.02
rHb-β- F41K	18.84 ± 0.36	1.69 ± 0.13	27.14 ± 0.73	1.79 ± 0.12

**Figure 13:**
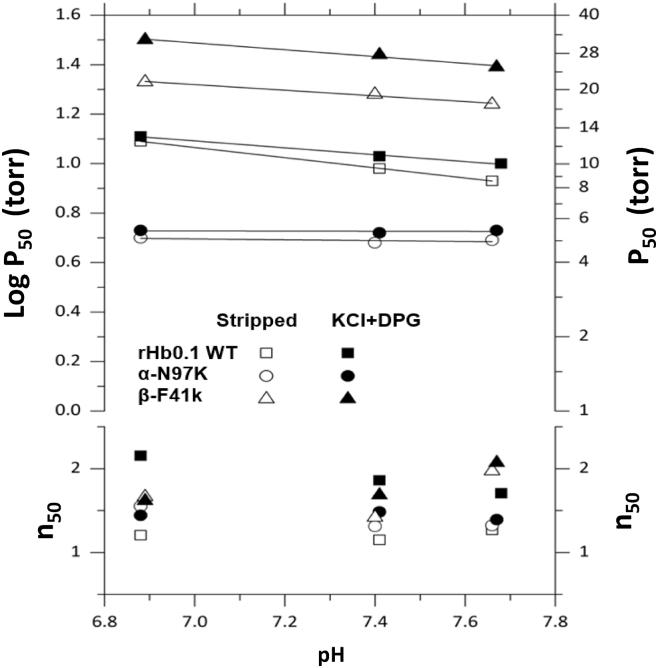
**Measurement of O_2_-binding affinity (P_50_) and Hill coefficient (n_50_) of recombinant human hemoglobin** wild-type (rHb0.1 WT), rHb-α-N97K, and rHb-β-F41K mutant proteins in the absence of allosteric effector (stripped) and presence of allosteric effector (KCl and DPG).

Oxygen binding to Hb is cooperative in nature, and it is measured by the Hill coefficient (*n*). RBC Hb (HbA) has an n_50_ value of approximately 2.8 to 3 (38, 39). rHb with low oxygen affinity (high P_50_) and high cooperativity represents requisite features for the development of viable HBOC. Outside the red blood cell, Hb-oxygen binding is not allosterically regulated by effector molecules such as 2,3-diphosphoglycerate (2,3-DPG) and Hb-oxygen affinity is therefore greatly increased, and cooperativity is correspondingly reduced. We, therefore, measured the oxygen-binding affinity and cooperativity of each mutant, as shown in Figure 13. The experiments revealed that rHb-α-N97K and rHb-β-F41K have cooperativities lower than rHb0.1 WT in presence of allosteric effectors (Table 4).

## Discussion

Attempts were made to develop Hb-based artificial oxygen carriers with limited success due to their poor stability and rapid heme dissociation that rendered toxicity to humans. These products were further modified by intramolecular chemical or genetic cross linking, polymerization, PEGylation, encapsulation, and conjugation with enzymatic reductant systems such as catalase or superoxide dismutase (40) to improve its various characteristics like stability, circulation half-life and autooxidation. We have employed protein engineering tools to overcome these challenges.

However, there is a need for efficient production systems for large scale development of these agents. During 1990s, Hoffman *et al.* had reported the first successful tandem expressions of α- and β-subunits to form complete and functional recombinant human hemoglobin. However, it is already reported that the α-subunit is difficult to express in *E. coli* because of the solubility issues in the absence of β-globins and a chaperone AHSP (22, 41, 42). The stability problem of unequal expression of Hb subunits was well-known long before recombinant production in heterologous host cells was developed. Similarly, we observed in SDS-PAGE profile that expression of di-alpha is less when compared to beta subunit, probably due to the absence of AHSP (alpha-hemoglobin stabilizing protein) in *E. coli*, a human chaperone protein present in erythroid cells that binds to the α-subunit before association to the β-subunit, thereby relieving the stability issue of individual α-subunits and aiding tetramer formation *in vivo* (43). The stoichiometry of α-chain (one linked subunit) to the β-chains (two subunits) and the difference in the molecular weights of these chains may also result in appearance of α-chain being less than β-chains. However, we have utilized all fractions displayed on the gel for subsequent experiments since the ratio of α-and β-chains remain consistent throughout the gel, and these fractions are entirely pure (Fig. 2*B*). (44, 45). Yet, to produce sufficient quantities of Hb for a hemoglobin-based oxygen carrier (HBOC), manufacturing would have to transition to large-scale fermentation.

For the successful development of an HBOC, the kinetics of heme release from rHb is a major consideration. Free heme can act as a DAMP molecule and activates Toll-like receptor 4 of the immune system resulting in inflammation, oxidant production, vascular injury and triggers oxidative reactivity itself (46). It is evident from previous studies that recombinant protein rHb3011 failed in clinical trials due to release of free heme and high rates of autooxidation (47). Ferric (met) form of Hb readily releases heme. A combination of heme extraction assay, reversed phase chromatography, pH and GuHCl induced denaturation studies clearly indicate the absence of any kind of covalent linkage between heme and protein in rHb-β-F41K due to release of free heme under different experimental conditions as mentioned earlier in result section.

To assess the functional impact of this substitution, we employed heme transfer kinetics to apomyoglobin, a well-established method previously used to quantify hemin dissociation from engineered Hbs (15, 48). Compared to rHb0.1WT, the β-F41K mutant exhibited ∼4-fold lower heme dissociation from the β-subunit and ∼3-fold lower from the α-subunit. When compared to native HbA (K_fast_ = 10.39 ± 0.1 h^−1^, K_slow_ = 0.9 ± 0.02 h^−1^ (30), β-F41K retained heme ∼2.6-fold better in the β-subunit and ∼2.5-fold better in the α-subunit. In the present study, the β-F41K mutation was specifically designed to enhance heme affinity in the β-subunit. In our kinetic analysis, we continued to observe a biphasic dissociation profile, with a distinct fast and slow phase. While the fast phase was substantially slower in β-F41K compared to rHb0.1WT, its presence and magnitude are still consistent with previous observations attributed to the heme loss from the β-subunit. Thus, although the rate is decreased, we interpret the persistence of a biphasic pattern with the fast phase remaining dominant as evidence that the β-subunit continues to be the primary contributor to the faster phase. Regarding the observation that the α-chain also appears more stable in this mutant, we hypothesize that this arises from an overall increase in tetramer stability or inter-subunit cooperativity. A comparative analysis (Table S3) highlights the superior heme affinity and physiologically relevant oxygen affinity of rHb-β-F41K relative to other engineered rHb variants. We hypothesize that the F41K mutation in β-chain alters the creation of αβ subunit connections, mitigating fluctuations in the globin tertiary structure. This alteration significantly decreases the entry of water into the heme pocket’s proximal region and hinders the hydration of the FeHis(F8) bond (49). Moreover, α-mutant Y42H demonstrated nearly two-fold better heme retention than rHb0.1WT but was not thermally as stable as rHb-β-F41K and had low expression yield. Few mutants like α-L101E and α-V132K did not follow the bipasic heme kinetics unlike other mutants, while beta N102E mutant exhibited similar k_slow_ as WT. In contrast to rHb-β-F41K, these rHb mutants exhibited low expression yield, which prevented further analyses. If mutants do not express well, we decided that their potential utility as artificial oxygen carriers were not worth investigating further.

The rates of autoxidation and hemin dissociation exhibited a significant dependence on the quaternary structure, increasing by over 10-fold when tetramers dissociate into dimers, and an additional 4- to 5-fold when monomers form. Therefore, employing genetic or chemical cross-linking to hinder dimerization should considerably diminish the rate of autoxidation and hemin loss compared to native HbA, particularly at low protein concentrations where dimerization is prevalent (14, 31, 50). In native HbA, α2β2 tetramers undergo reversible dissociation into αβ dimers through bond cleavage at the α1β1 and α2β1 interfaces and the equilibrium dissociation constant K_(4,2)_ = [Dimer]^2^/[Tetramer] is approximately 1 μM for liganded Hb, indicating a greater propensity for dissociation compared to deoxy tetramers (51). To accurately characterize the properties of the Hb tetramer, it is crucial to consider the presence and contribution of dimers within the Hb solution. In order to investigate whether the mutations introduced into the rHb0.1 prototype could impact the Hb tetramer dissociation equilibrium at the minimal protein concentration used during heme loss kinetics, we performed size-exclusion chromatography. Interestingly, our findings imply that none of the mutant underwent tetramer dissociation into αβ dimers (including the heme stabilized mutant rHb-β-F41K), which could be due to the presence of glycine linker between C terminal of Arg-α1 and N-terminal of Val-α2 in rHb0.1.

Oxy rHb or ferrous rHb spontaneously autooxidizes to ferric Hb under air-saturated conditions. In these experiments, we observed a constant decline in the absorbance at 541 and 576 nm along with a corresponding increase in the absorbance at 630 nm, both of which characterize the formation of metHb (52). For rHb0.1 WT and rHb-β-F41K, there was little or no change in the absorbance at 700 nm, which showed that there was no precipitation of the protein during the period of incubation (10 h). It is also interesting to note that the other mutants with better heme affinity than rHb0.1WT like rHb-α-Y42H, rHb-β-S44H/β-N102K, rHb-β-S44H/α-N97H, rHb-α-L66H/α-N97K displayed autooxidation rates similar to rHb0.1 WT (Fig. S2), though they had poor stability and protein expression yields. The inclusion of the glycine linker between the α-polypeptides in our design was intended to stabilize the hemoglobin tetramer and prevent dissociation into dimers, a key limitation of unmodified hemoglobin in HBOC development. The β-F41K mutation was introduced to modify the heme pocket environment, enhancing heme retention while maintaining oxygen-carrying capacity. The 2-fold higher auto-oxidation rates observed in our mutant compared to OptroTM are likely due to changes in the heme pocket environment caused by the β-F41K mutation (30). Structural alterations near the heme iron can increase susceptibility to oxidative stress. However, we note that our mutant shows a three to four-fold improvement in heme retention of the β-subunits compared to OptroTM, which is a significant advantage in reducing heme toxicity and ensuring long-term stability of the tetramer. We acknowledge that balancing oxidative stability with heme retention is a challenge in HBOC design. Our findings suggest that while β-F41K confers superior heme retention, further optimization may be needed to improve the auto-oxidation rates, though its native like oxygen affinity is a distinct advantage. Future work will explore combinations of stabilizing mutations and other approaches to mitigate oxidation while maintaining the benefits of improved heme retention and oxygen affinity.

When it comes to the development of HBOCs, the potential thermal stability of cross-linked hemoglobin is critically assessed. This assessment helps determine whether the distinctive structure and functions of the protein can be maintained even under higher temperatures (53). Frequently, thermal energy exacerbates the destabilization of Hb by disrupting hydrophobic interactions, hydrogen bonds, and the heme component. This leads to the aggregation and precipitation of the protein (54). Interestingly, Phe to Lys mutation at C7 position in beta subunit markedly stabilizes overall Hb structure from any precipitation up to 62 °C, an apparent T_m_ predicted with the help of circular dichroism spectroscopy. This evidence suggests that Lys may exhibit some intramolecular interaction with the heme that helps to fasten the α1β2 interface *via* intermolecular interactions resulting in the requirement of higher thermal energy to melt it. Although the β- S44H/β- N102K double mutant exhibited thermal stability similar to β-F41K, with T_m_ of 62 °C, it exhibited a lower heme stability relative to rHb-β-F41K. Conversely, mutants like α-Y42H, β-S44H/α-N97H, and α-L66H/α-N97K had better heme retention than rHb0.1WT but displayed lower thermal stability relative to rHb-β-F41K, exhibiting T_m_ in the range of 50 to 55 °C; rHb-β-F41K was thus the only mutant with all the desired characteristics.

The vital characteristic of a prototype of HBOC is its oxygen affinity (P_50_), which needs to be in the range of oxygen affinity of Hb in red blood cells (55, 56, 57). As such, we did not investigate the P_50_ and Hill coefficient of α-Y42H, α-L101E, α-V132K, β-N102E, β-S44H/α-N97H, α-L66H/α-N97K, and β- S44H/β- N102K due to the unfavorable characteristics discussed above that do not warrant further investigation as potential HBOCs. The P_50_ for the mutant rHb-β-F41K, on the contrary, was measured since it exhibited all the favorable characteristics, namely, thermal stability, expression yield, and heme retention, along with its slightly slower auto-oxidation rate compared to the wild-type protein under physiological conditions; the other mutants are deficient in one or another property. The O_2_-binding properties of HbA, rHb0.1 WT and rHb-β-F41K are presented in the Table 4. Data for rHb-α-N97K, which showed high O_2_ affinity, was included just for comparison. Although rHb-α-N97K variant was not part of the earlier oxidative stability assays due to its faster heme dissociation kinetics, we were able to determine its oxygen affinity. The higher affinity observed in α-N97K compared to Hb0.1 likely result from its position within the α_1_β_1_ interface, where substitution at N97 may influence intersubunit contacts and slightly shift the allosteric equilibrium toward the R-state. Because T to R state transitions are mediated largely through α_1_β_2_ interactions, high affinity variants frequently result from substitutions that alter this interface (58).

Figure 12 demonstrates clearly that the replacement of the β-subunit C7 Phe with Lys had significantly changed the O_2_ binding properties of the macromolecule. Surprisingly, rHb-β-F41K displayed O_2_ affinity lower than rHb0.1WT but was functionally nearly identical to HbA, with P_50_ similar to Hb in red blood cell. The Hill coefficients (n50), an indicator of cooperativity in the oxygenation process of Hb, are plotted as a function of pH in Figure 13. As summarized in Table 4, the Hill coefficients at pH 7.4 (in the presence of allosteric effectors) for HbA, rHb0.1WT, rHb-α-N97K and rHb-β-F41K are 2.7, 1.91 ± 0.03, 1.44 ± 0.02 and 1.79 ± 0.12, respectively. The rHb0.1WT and mutant rHb-β-F41K showed lower value than those of HbA, presumably due to the presence of the initiator methionine or an increased rate of autoxidation (59, 60, 61, 62). The β-F41K mutant (P_50_ = 27 torr) maintains a low level of cooperativity, making it a promising candidate for artificial oxygen carriers. Its oxygen affinity closely resembles that of native RBC hemoglobin, ensuring effective oxygen loading and unloading. In contrast, Optro (P_50_ = 34 mmHg) (30) may release oxygen too readily, while rHb-β-T84Y (P_50_ = 7.16 torr) binds oxygen too tightly, potentially limiting tissue oxygen delivery (63). Hemolink (P_50_ = 38–40 mmHg) has even lower oxygen affinity and a non-cooperative binding profile (Hill coefficient = 1 *versus* 2.8 for native Hb), which may affect its oxygen transport efficiency (30). These comparisons highlight β-F41K’s balanced oxygen-binding properties, making it a promising HBOC candidate. In contrast to previously developed low-oxygen-affinity rHbs such as rHb(β-N108Q), which involve substitutions at the subunit interface and central cavity to modulate allosteric regulation and reduce autoxidation (64), the β-F41K mutation targets a heme-proximal site within the β-subunit to enhance heme retention with moderate oxygen affinity comparable to HbA. While rHb(β-N108Q) demonstrated improved redox stability and altered oxygen-binding properties (lower oxygen affinity than HbA) (64) (Table S3), the rHb-β-F41K mutant maintained a P_50_ similar to native HbA and exhibited a slightly slower autooxidation rate compared to the wild-type rHb0.1. These results suggest that β-F41K stabilizes the heme environment *via* local interactions rather than quaternary structural alterations, offering a complementary route to improving recombinant Hb stability without compromising oxygen delivery characteristics. In subsequent investigations, the structural and molecular basis underlying the enhanced heme retention and RBC-like oxygen affinity exhibited by the β-F41K mutant will be further explored in addition to its potential role in nitric oxide (NO) scavenging. Although further improvements may be attempted, the current developments are significant for the recombinant Hb (β-F41K) to function as a HBOC prototype. The prototype developed here exhibits a remarkably enhanced heme affinity, greater than any previously reported Hb mutant. It also exhibits sufficient stability for long-term storage and possesses optimal autooxidation characteristics. Importantly, its oxygen-binding properties closely resemble those of native red blood cell Hb, making it well-suited for use as a blood substitute in short-term applications. Building on these promising functional properties, efforts will be directed toward scaling up the production of this recombinant hemoglobin variant to facilitate comprehensive preclinical studies focused on evaluating its safety and efficacy in animal models.

## Conclusion

The attempt at engineering rHb0.1 to confer higher heme stability accompanied by physiologically relevant oxygen affinity, as presented in this investigation, was largely successful. Heme release kinetics are crucial for HBOC development, with free heme posing significant toxicity risks. Mutant rHb-β-F41K exhibited substantially slower rates of heme loss compared to rHb0.1 WT, suggesting a potential solution to this challenge. The mutation likely alters αβ subunit connections, reducing fluctuations in globin tertiary structure and hindering water entry into the heme pocket. However, the other mutants exhibited varied results, with some showing promising heme retention but inadequate thermal stability or expression yield. The rates of autoxidation and hemin dissociation are influenced by quaternary structure, emphasizing the importance of preventing tetramer dissociation into dimers. Genetic cross-linking strategies mitigated this issue, reducing autoxidation and hemin loss; the di-α cross link in rHb-β-F41K served this purpose in conjunction with the mutation itself. Notably, rHb-β-F41K demonstrated a lower oxygen affinity than rHb0.1 WT but maintained functional properties like HbA, with oxygen affinity similar to Hb inside red blood cells, a hallmark of a successful HBOC prototype. The mutant retains a degree of cooperativity making it a viable candidate for an artificial oxygen carrier. Addressing challenges in large-scale production, the mutant gained enhanced stability, crucial for the successful development of HBOCs. The novel mutant rHb-β-F41K showed promise in improving heme affinity while maintaining functional integrity, offering potential solutions for the advancement of development of artificial oxygen carriers. Further research and optimization efforts are warranted to realize the full potential of protein-based oxygen carriers.

## Experimental procedures

### Site-directed mutagenesis

The gene for recombinant human hemoglobin wild type (rHb0.1) subcloned into pET28a plasmid vector using BamHI and HindIII restriction enzymes was obtained as a kind gift from Prof. John S. Olson, Rice University. The construct contains di-alpha gene cross-linked with a glycine linker and one beta gene with N-terminal 6X His tag. This was used as a template for the generation of mutants using the Quik Change site-directed mutagenesis Kit (Agilent Technologies Inc). To incorporate the desired mutations in the plasmid, mutagenic oligonucleotide primers were designed as per instructions of the manufacturer, and HPLC purified primers were synthesized (Eurofins Genomics Pvt. Ltd). PCR conditions employed for the site-directed mutagenesis method were as follows: initial denaturation at 95 °C for 5 min, 21 cycles of 95 °C for 30 s, 55 °C for 1 min 30 s, 72 °C for 10 min and final extension at 72 °C for 12 min. The PCR product was then digested overnight at 25 °C with DpnI to remove the methylated circular DNA.

### Plasmid isolation

10 μl of the digested product was then transformed into DH5α competent cells. The resulting colonies were inoculated in 5 ml of LB media and cultured overnight. Plasmid isolation was performed for rHb0.1 and its mutant clones using a Gen Elute Plasmid Mini-Prep kit from Sigma. The isolated plasmid was verified by electrophoresis on agarose gel and mutations were confirmed by DNA sequencing through AgriGenome Pvt. Ltd.

### Expression of recombinant protein

*E. coli* BL21(λDE3) (Invitrogen) cells were transformed with pET28a.rHb0.1 or its different mutants *i.e.*, plasmid vectors containing the genes for different mutants of Hb under investigation. Single isolated colonies were used for inoculation of Terrific Broth (TB) (HiMedia) media. Cells were allowed to grow to an absorbance of approximately 0.6 to 0.8 (at 600 nm) at 37 °C for 8 to 10 h. For rHb0.1 and its mutant proteins, expression was performed at 30 °C at 200 rpm speed for 14 h in an incubator (Eppendorf, New Brunswick Innova 44, Swedesboro). The cells were harvested by centrifugation at 7000 x g at 4°C for 10 min.

### Purification of recombinant proteins

The harvested cells were suspended in a lysis buffer (3 ml/gm of cell pellet). Lysis buffer contained 100 mM potassium phosphate buffer pH 7.4, 100 mM NaCl, and 1 mM PMSF (BioBasic Inc. Konrad Cr). After resuspension, 300 μg of lysozyme (BioBasic Inc. Konrad Cr) per gm of cell pellet was added and incubated at room temperature for 30 min with mild agitation. Finally, 30 μg of DNase (BioBasic Inc. Konrad Cr) per gm of cell pellet was added and incubated for an additional 25 min at room temperature with constant mild shaking. The lysed cell pellet was stored at −20 °C. When required, the pellets were freeze-thawed and subjected to sonication using W-385 Heat Systems (Ultrasonics Inc.). Sonication conditions were: 45% amplitude, 10s ON and 30s OFF for 3 min. This was followed by centrifugation at 17,000*g* at 4 °C for 45 min (RC 5B SORVALL, Thermo Fisher Scientific). Purification was carried out by Ni-NTA agarose (GE Healthcare) affinity chromatography. Equilibration of the column was achieved using a binding buffer 100 mM potassium-phosphate buffer, 100 mM NaCl, 5 mM imidazole (Hi-Media), pH 7.4. The cell extract obtained after sonication was loaded on Ni-NTA agarose for binding. After binding, unbound proteins were washed twice with binding buffer. Elution of protein was achieved using a binding buffer containing 100 mM imidazole. The purified His-tagged protein was then loaded on Sephacryl S-200 (GE Healthcare) based gel filtration chromatography equilibrated with 100 mM potassium-phosphate buffer, pH 7.4. The rHb0.1 and mutant proteins were then subjected to reduction and oxidation by sodium dithionite (Merck Life Sciences Pvt Ltd. Delhi, India) and potassium ferricyanide (Merck Life Sciences Pvt Ltd. Delhi, India) respectively, following desalting on Sephadex G-25 (GE Healthcare) column. Abs_Soret_/Abs_280nm_ absorbance ratio of 3.5 and higher indicated the purity of proteins, which was additionally confirmed by 15% SDS-PAGE after staining with silver nitrate. Pure protein was stored at −80 °C for further use. The expression and purification procedure for mutant proteins were performed similarly as for the wild-type protein.

### Spectroscopic analysis

#### UV-visible absorbance spectroscopy

Absorbance measurements were carried out using Cary Varian Bio100 UV-Vis spectrophotometer attached to a Peltier thermostat (Varian Inc). UV-Vis spectra were recorded in the wavelength range of 200 to 700 nm. Quartz cuvettes were used for the measurements (27).

#### Circular dichroism spectroscopy

CD spectra were recorded on a JASCO J-815 spectropolarimeter (JASCO Corporation) using a cylindrical quartz cell of path length 1 mm for far- UV region. The changes in the secondary structure of the protein were monitored in the far- UV region between 190 to 260 nm. Three consecutive spectral scans were averaged and corrected by subtracting corresponding blanks and subjected to noise reduction.

### Heme extraction assay

The rHb0.1 WT and mutant proteins were assessed for their heme retention abilities using a heme extraction assay following Teale’s method (65). Briefly, the pH of Hb samples was lowered to pH 2.0 using 0.1 M HCl (ice chilled) (Qualigens) followed by the addition of an equal volume of cold butanone (SD Fine Chemical Limited). The samples were then mixed vigorously to separate the two phases, the top organic phase containing the heme while the bottom aqueous phase containing the resulting apo-globin.

### Measurement of heme dissociation kinetics

The rates of heme dissociation of rHb0.1 protein was measured as described previously (14, 48). Briefly, the transfer of hemin from 3 μM of ferric rHb (holo) protein in 0.1 M potassium phosphate buffer, pH 7.4 containing 0.45 M sucrose to 30 μM H64Y/V68F apo-myoglobin was measured by monitoring the ratio of absorbance at 410 nm and 600 nm. The assay was performed for 300 min at 37 °C and measured by following the decrease in absorbance at 410 nm as “green” holo-metmyoglobin (H64Y/V68F) was formed, which absorbs at 600 nm. The normalized absorbance changes were calculated by taking the ratio of A_410nm_/A_600nm_ and plotted against time (min). Data were fitted to two-phase exponential equation using the GraphPad Prism 5 (GraphPad Software, Inc, San Diego, CA) (66) and analyzed as the average of three independent experiments.

### Quaternary structure determination by size exclusion chromatography

Size exclusion chromatography was performed using Superdex 75 10/300 Gl column (GE Healthcare Life Sciences). 1 ml of protein sample at a concentration of 15 to 20 mg/ml was injected using a 500 μl injection loop on an AKTA start (Cytiva). UV–Vis detector was used to monitor the protein elution. 100 mM phosphate buffer (100 mM NaCl, pH 7.4) was used to equilibrate the column. The sample was analyzed at a flow rate of 1 ml/min. The elution of protein was monitored by measuring absorbance at 280 nm in the chromatogram. Control protein HbA (native human hemoglobin) was used to estimate the molecular weight of rHb0.1 WT and rHb-β-F41K.

### Quaternary structure analysis of mutants at minimal concentration needed for heme dissociation kinetics

Size exclusion chromatography was conducted at ambient temperature using an HPLC system from Waters (Model 2489; Milford). Protein elution was monitored with a UV–vis detector. The column (BioSep 5 μm SEC-s2000 145 Å, LC Column 300 × 4.6 mm; Phenomenex Inc) was equilibrated with 100 mM phosphate buffer of pH 7.4. A 20 μl protein sample at a concentration of 3 μM heme was injected and analyzed at a flow rate of 1 ml/min. Protein elution was tracked by measuring absorbance at 280 nm in the chromatogram. Marker proteins Catalase and Trypsin (Sigma Aldrich); Lysozyme (BioBasic Inc Konrad Cr); Conalbumin (GE Healthcare) and rHb0.1WT at 300 μM concentration were employed to estimate the molecular weight of rHb mutant proteins at 3 μM concentration used for heme loss kinetics by generating a standard curve plotting logarithmic values of molecular weight against elution volume.

### pH stability measurement to compare structural alteration between rHb0.1WT and rHb-β-F41K

To examine pH-associated structural modifications and pH-dependent stability, buffers were prepared across a pH range of 2.0 to 11.0 as follows: 100 mM glycine-HCl for pH 2 to 3.5; 100 mM sodium acetate for pH 4.0 and 4.5; 100 mM potassium phosphate for pH 5.0 to 8.0; and 100 mM borate-boric acid or sodium hydroxide for pH 8.5 to 11.0. The purified proteins were diluted in the respective pH buffer to a final concentration of approximately 0.15 mg/ml, followed by incubation for 3 to 4 h at 25 °C. Absorbance spectra were then measured in the ranges of 260 to 700 nm. Data is an average of three independent experiments.

### Thermal stability measurements

The thermal stability of the purified proteins was monitored in the range of 20 to 90 °C as described earlier (29). Protein samples were diluted in 100 mM phosphate buffer with a pH of 7.0 to achieve a concentration of approximately 0.1 mg/ml. Circular dichroism spectroscopy (JASCO Corporation) was employed to examine the impact of temperature on the stability of both rHb0.1 and its mutants. The unfolding curves provide global insight into thermal denaturation of proteins and help in suitable calculation of T_m_ (transition thermal mid-point), a quantitative indicator of stability and denaturation. Data is an average of three independent experiments.

### Secondary structural analysis of rHb-β-F41K in the presence of chaotropes using CD spectroscopy

pH induced denaturation of the proteins was carried out using different buffers across a pH range of 2.0 to 11.0 as described in Thermal stability measurements. The purified proteins were diluted in the respective pH buffer to a final concentration of approximately 0.15 mg/ml, followed by incubation for 3 to 4 h at 25 °C. For GuHCl stability studies, the pure protein was diluted in various concentrations of GuHCl (Sisco Research Laboratories Pvt. Ltd, Maharashtra, India) (0–6.0 M) at pH 7.0 and incubated for 3 to 4 h. The protein concentration used for stability studies was approximately 0.15 mg/ml. CD spectra was then measured in the ranges of 260–190 nm. The % helical content was calculated manually by measuring the change in ellipticity at 222 nm between folded and unfolded states, normalized to the native state. Data is an average of three independent experiments.

The calculation was performed as follows:

% Helicity = [(Ellipticity at 222 nm (native) − Ellipticity at 222 nm (denatured))/Ellipticity at 222 nm (native)] × 100.

### Autooxidation measurements

Autooxidation rates of wild-type and mutant rHb0.1 protein were measured by monitoring the changes in absorbance at 576 nm and 630 nm as described earlier (36). Briefly, 50 μM oxy-protein in 50 mM potassium phosphate buffer (pH 7.4), containing 1 mM EDTA (Thermo Fisher Scientific Pvt Ltd) and 3 mmol/mol of heme catalase (Sigma) and superoxide dismutase (Sigma). The oxidation of the protein samples was monitored by measuring the decrease in absorbance at 576 nm in scanning kinetics mode for a period of 10 h at 37 °C using Cary Varian 100 UV-visible spectrophotometer. Data were fitted to a single exponential equation using the GraphPad Prism 5 (GraphPad Software) (66) and analyzed as the average of three independent experiments.

### Hydrogen Peroxide-mediated oxidation

The reaction of ferrous hemoglobin (Hb) with a 10-fold molar excess of H_2_O_2_ (1:10) was conducted using a photodiode array spectrophotometer. Hb samples were incubated with H_2_O_2_ for 5 min, followed by the addition of catalase (200 units/ml) for 1 min to ensure complete removal of H_2_O_2_. Ferryl Hb formation was detected using a previously described method, which involved adding 2 mM sodium sulphide (Na_2_S) to the reaction mixture. This converts ferryl Hb to sulfhemoglobin (sulfHb). Fresh stock solutions of Na_2_S were prepared for each experiment (30). The levels of ferryl Hb were quantified by measuring the absorbance of sulfHb at 620 nm, using its extinction coefficient.

### Measurement of oxygen binding properties

Purified rHb0.1 and its mutants were mixed with sodium dithionite (1 mg/ml; Sigma-Aldrich, Inc.) and immediately passed through a PD-10 desalting column (GE Healthcare) equilibrated with 25 ml of 0.01 M HEPES/0.5 mM EDTA (pH 7.4) (Sigma-Aldrich, Inc). Eluates were concentrated using Amicon Ultra-4 Centrifugal Filter Units (Millipore). Sample concentration was measured spectrophotometrically using published extinction coefficients (67). The oxygen binding parameter of recombinant human hemoglobin wild type and mutant protein solutions (0.25 mM Hb) were measured on a Blood Oxygen Binding System (Loligo Systems) (BOBS) at 37 °C as a function of pH in 0.1 M HEPES/0.05 M EDTA buffer. The BOBS is a gas diffusion chamber capable of equilibrating samples to various partial pressures of O_2_, CO_2_ and N_2_ while constantly monitoring sample light absorption (190–850 nm) with the built-in spectrometer. This unit can produce Hb-O_2_ equilibrium curves based on a modified version of the thin film technique developed by Weber (1992). This technique has the advantage of greatly reduced samples sizes relative to other instruments, as well as full control and flexibility of experimental conditions (buffer type, temperature and PCO_2_). O_2_-equillibrium curves were measured in the absence (stripped) and presence of chloride ions (100 mM KCl) and organic phosphates (0.5 mM 2,3-diphosphoglycerate; Sigma-Aldrich, Inc.). Each Hb solution was sequentially equilibrated with three to five different oxygen tensions (*P*_O2_) at saturation levels between 30 to 70% while the absorbance was continually monitored at 430 nm (deoxy peak) and 421 nm (oxy/deoxy isosbestic point). Hill plots (log [fractional saturation/[1-fractional saturation] vs log*P*_O2_) constructed from these measurements were used to determine the *P*_O2_ at half saturation (*P*_50_) and the cooperativity coefficient (*n*_50_) from the χ-intercept and slope of these plots, respectively. *P*_50_ values were measured at three different pH levels, where the pH of working solutions was adjusted with NaOH to as near 7.2, 7.4, or 7.6 as possible, then precisely measured with an Orion Star A211 pH Meter and Orion PerpHecT ROSS Combination pH Micro Electrode (Thermo Fisher Scientific). A linear regression was fit to plots of log*P*_50_ *versus* pH and the resulting equation was used to estimate *P*_50_ values at pH 7.40 (± SE of the regression estimate). Data are an average of three independent experiments.

### Graphical representation and analysis

Graphs for the thermal induced denaturation experiments obtained from spectral analysis were plotted using the GraphPad Prism 5 (GraphPad Software, Inc) (66).

### *In-silico* structural analysis

All protein structures were visualized and analysed using PyMol, UCSF Chimera, and SWISS PDB viewer software (25, 68, 69).

## Data availability

All data are available in the main text or the supporting information and supporting tables.

## Supporting information

This article contains supporting information (15, 27, 29, 30, 64).

## Conflict of interest

The authors declare that they have no conflicts of interest with the contents of this article.
