# Supplementary material for: Hemoglobin β-F41K: A recombinant oxygen carrier prototype engineered for enhanced heme retention, stability, and optimal oxygenation properties
Source: J Biol Chem. 2025 Jul 11;301(8):110477. doi: 10.1016/j.jbc.2025.110477 (PMC12355045; doi:10.1016/j.jbc.2025.110477)
Supplement: Supporting information [file mmc1.docx]

**Supplementary Information**

**for**

**Hemoglobin β-F41K: a recombinant oxygen carrier prototype engineered for enhanced heme retention, stability, and optimal oxygenation properties**

Mohd Asim Khan^aµ@^, Kajal Yadav^a@^, Anthony V. Signore^b^_,_ Nidhi Mittal^a$^, Sheetal Uppal^a#^, Amit K. Tyagi^c^, Gaurav Mittal^c^, Jay F. Storz^b^ and Suman Kundu^a,d^*

^a^ Department of Biochemistry, University of Delhi South Campus, New Delhi-110021, India.

^b^ School of Biological Sciences, University of Nebraska, Lincoln, NE-68588, USA.

^c^Institute of Nuclear Medicine and Allied Sciences, Defence Research and Development Organization, Delhi-110054, India.

^d^Department of Biological Sciences, Birla Institute of Technology and Science Pilani, K K Birla Goa Campus, Goa-403726, India.

**Running Title:** Engineering enhanced heme retention in hemoglobin

To whom correspondence should be addressed: Suman Kundu, Department of Biochemistry, University of Delhi South Campus, New Delhi, India and Department of Biological Sciences, Birla Institute of Technology and Science Pilani, K K Birla Goa Campus, Goa, India; Tel: +91-832-2580101; E-mail: [suman.kundu@south.du.ac.in](mailto:suman.kundu@south.du.ac.in); skundu@goa.bits-pilani.ac.in

^@^Equal contribution

Note – ^µ^ M.A.K. is presently working in William G. Lowrie Department of Chemical and Biomolecular Engineering, The Ohio State University, Ohio 43210, USA.

^$^ N.M. is presently working in Molecular Biophysics Unit, Indian Institute of Science, Bangalore 560012, India.

^#^ S.U. is presently working in National Eye Institute, National Institutes of Health, Bethesda MD 20892, USA.

**Table S1 A. Mutants generated in recombinant hemoglobin (rHb0.1) α- and β- chains**

| **S.No** | **α-chain** | **Helix** | **S.No** | **β-chain** | **Helix** |
| --- | --- | --- | --- | --- | --- |
| 1. | T39H | C4 | 1. | F41H | C7 |
| 2. | Y42H | C7 | 2. | F42H | CD1 |
| 3. | F43H | CD1 | 3. | S44H | CD3 |
| 4. | K61H | E10 | 4. | K66T | E10 |
| 5. | V62H | E11 | 5. | K66H | E10 |
| 6. | L66H | E16 | 6. | V67H | E11 |
| 7. | N97H | G4 | 7. | F71H | E15 |
| 8. | L101H | G8 | 8. | N102H | G4 |
| 9. | S102H | G9 | 9. | L106H | G8 |
| 10. | L105H | G12 | 10. | G107H | G9 |
| 11. | S131H | H14 | 11. | L110H | G12 |
| 12. | V132H | H15 | 12. | V137H | H15 |
| 13. | S133H | H16 | 13. | A138H | H16 |
| 14. | L136H | H19 | 14. | L141H | H19 |
| 15. | Y42K | C7 | 15. | F41K | C7 |
| 16. | N97K | G4 | 16. | N102K | G4 |
| 17. | L101K | G8 | 17. | F41E | C7 |
| 18. | V132K | H15 | 18. | V67E | E11 |
| 19. | L136K | H16 | 19. | A70E | E14 |
| 20 | M32E | B13 | 20. | N102E | G4 |
| 21. | K61E | E10 | 21. | L106E | G8 |
| 22. | A65E | E15 | 22. | L141E | H19 |
| 23. | N97E | G4 | 23. | F41D | C7 |
| 24. | L101E | G8 | 24. | V67D | E11 |
| 25. | L136E | H19 | 25. | A70D | E14 |
| 26. | A65D | E15 | 26. | L141D | H19 |
| 27. | N97D | G4 |  |  |  |
| 28. | L136D | H19 |  |  |  |

## Table S1 B. Combination of mutants generated in recombinant hemoglobin (rHb0.1) α- and β-chains

| **S.No** | **Amino acid mutation** |
| --- | --- |
| 1. | α-V132H/α-L101H |
| 2. | β-S44H/α-N97H |
| 3. | β-S44H/α-L101K |
| 4. | β-S44H/β-N102K |
| 5. | β-S44H/β-K66H |
| 6. | β-S44H/β-K66T |
| 7. | β-S44H/α-N97K |
| 8. | β-S44H/α-L66H |
| 9. | α-L66H/α-N97K |
| 10. | α-N97K/β-N102K |
| 11. | β-S44H/β-N102K/α-N97K |
| 12. | α-N97K/ α-L136K/β-S44H/β-N102K |

## Table S2. The rates of heme dissociation in rHb0.1 WT and its mutant proteins, along with the percentage of fast versus slow dissociation are listed.

| S.no. | Protein | Heme transfer rate (h^-1^)  k _slow_ (h^-1^) k _fast_ (h^-1^) | |
| --- | --- | --- | --- |
|  | rHb0.1 WT | 0.96 ± 0.0005 | 16.2 ± 0.013 |
|  | α-M32E | 0.24 ± 0.001 (75% slower) | 15.6 ± 0.02 (3.7% slower) |
|  | α-T39H | 1.8 ± 0.012 (87.5% faster) | 17.76 ± 0.05 (9.6% faster) |
|  | α-Y42H | 0.48 ± 0.003 (50% slower) | 9.6± 0.01 (40% slower) |
|  | α-F43H | 1.26 ± 0.007 (31.2% faster) | 5.04 ± 0.009 (68.8% slower) |
|  | α-K61E | 0.96 ±0.005 (no change) | 7.2 ± 0.001 (55.5% slower) |
|  | α-N97K | 0.72 ± 0.003 (25% slower) | 16.2 ± 0.05 (no change) |
|  | α-N97H | 1.98 ± 0.001 (106% faster) | 27.6 ± 0.002 (70.3% faster) |
|  | α-N97D | 0.96 ± 0.0006 (no change) | 13.2 ± 0.021 (18.5% slower) |
|  | α-L101E | Monophasic kinetics; 3.06 ± 0.002 | |
|  | α-L101K | 1.26 ± 0.001 (31.2% faster) | 9.9 ± 0.018 (38.8% slower) |
|  | α-V132K | Monophasic kinetics; 2.4 ± 0.002 | |
|  | α-L136H | 0.78 ± 0.002 (18.7% slower) | 10.98 ± 0.02 (32.2% slower) |
|  | α-L136E | 1.8 ± 0.009 (87.5% faster) | 21.6 ± 0.012 (33.3% faster) |
|  | β-F41K | 0.36 ± 0.003 (62.5% slower) | 4.8 ± 0.037 (70.3% slower) |
|  | β-F41D | 0.96 ± 0.001 (no change) | 7.2 ± 0.02 (55.5% slower) |
|  | β-F41E | 0.48 ± 0.002 (50% slower) | 11.1 ± 0.013 (31.4% slower) |
|  | β-F42H | 1.2 ± 0.001 (25% faster) | 10.2 ± 0.01 (37% slower) |
|  | β-S44H | 1.02 ± 0.001 (6.2% faster) | 15.6 ± 0.02 (3.7% slower) |
|  | β-K66H | 2.64 ± 0.004 (175% faster) | 9 ± 0.002 (44.4% slower) |
|  | β-K66T | 0.78 ± 0.005 (18% slower) | 11.4± 0.02 (29.6% slower) |
|  | β-F71H | 1.8 ± 0.008 (87.5% faster) | 21.6 ± 0.08 (33.3% faster) |
|  | β-N102E | 0.84 ± 0.001 (12.5% slower) | 3.6 ± 0.07 (77.7% slower) |
|  | β-L110H | 0.96 ± 0.0005 (no change) | 16.2 ± 0.013 (no change) |
|  | β-L141H | 1.2 ± 0.008 (25% faster) | 6.6 ± 0.03 (59.2% slower) |
|  | β-L141D | 0.6 ± 0.002 (37.5% slower) | 17.4 ± 0.04 (7.4% faster) |
| 27. | α-L66H/ α-N97K | 0.48 ± 0.001 (50% slower) | 7.2 ± 0.003 (55% slower) |
| 28. | β- S44H/ α-L101K | 1.14 ± 0.003 (15.7% faster) | 3 ± 0.016 (81.4% slower) |
| 29. | β-S44H/ α-N97H | 0.54 ± 0.001 (43.7% slower) | 7.8 ± 0.01 (52% slower) |
| 30. | β- S44H/ β- N102K | 0.30 ± 0.005 (68.7% slower) | 12 ± 0.02 (25.9% slower) |
| 31. | α-N97K/ β- N102K | 1.08 ± 0.003 (12.5% faster) | 7.8 ± 0.04 (51.8% slower) |
| 32. | β- S44H/ α-L66H | 1.38 ± 0.004 (43.7% faster) | 9 ± 0.04 (44.4% slower) |

**Table S3: Rates of heme transfer, oxygen affinities and cooperativity of rHb variants compared to HbA**

| **Hemoglobin** | **Heme transfer rate**  **k_fast_ (h^-1^) k_slow_ (h^-1^)** | | **P_50_ (mmHg)** | **n_50_** |
| --- | --- | --- | --- | --- |
| HbA | 10.39± 0.1 ^a^ | 0.9± 0.02 ^a^ | 26.3 | 2.7 |
| rHb1.1 ^a^ | 7.20±0.45 | 0.30±0.01 | 35.00± 2.80 | 1.92± 0.91 |
| rHb-β-K82D(0.1 Prov) ^b^ | 6.1± 0.8 | 0.45± 0.04 | 14± 0.7 | 1.9 |
| rHb-β-N108K (0.1Pres)^b^ | 7.2± 3.4 | 0.30± 0.02 | 35± 2.8 | 1.6 |
| rHb-β-N108Q | - | - | 17.46 ^c^ | 3.10 ^c^ |
| rHb0.1WT | 16.2± 0.013 | 0.96 ± 0.0005 | 10.95 ± 0.10 | 1.91±0.03 |
| rHb-β-F41K | 4.8 ± 0.037 | 0.36 ± 0.003 | 27.14 ± 0.73 | 1.79±0.12 |

^a^ taken from (30) Meng, F., et al., Comprehensive Biochemical and Biophysical Characterization of Hemoglobin Based Oxygen Carrier Therapeutics: All HBOCs Are Not Created Equally. Bioconjug Chem, 2018. 29(5): p. 1560-1575.

^b^ taken from (15) Strader, M.B., et al., Engineering oxidative stability in human hemoglobin based on the Hb providence (βK82D) mutation and genetic cross-linking. Biochem J., 2017. 474(24): p. 4171-4192.

^c^ taken from (64) Tsai, C.-H., et al., Novel recombinant hemoglobin, rHb (βN108Q), with low oxygen affinity, high cooperativity, and stability against autoxidation. Biochemistry., 2000. 39(45): p. 13719-13729.


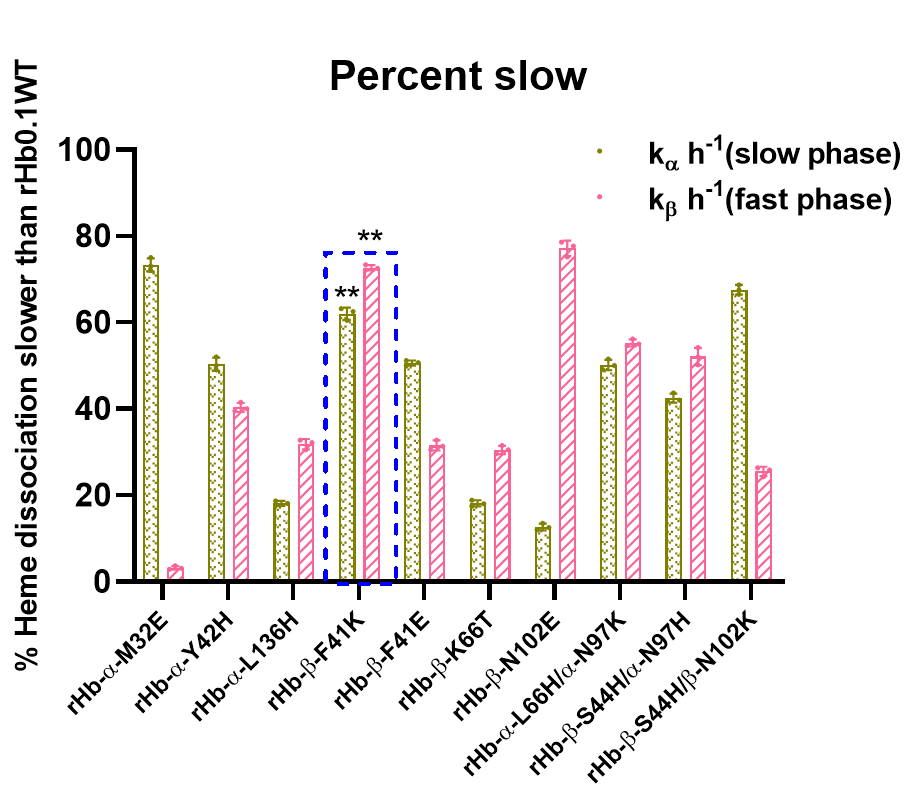


**Figure S1. The percentage of heme dissociation from mutants with slow heme release was compared to that of rHb0.1 WT.** Rates of slow and fast phase heme dissociation kinetics, denoted as kα and kβ (h^-1^), respectively, were determined for the mutants relative to rHb0.1 WT. Notably, rHb-β-F41K exhibited the highest percentage of slow heme release from both subunits compared to other mutants. The data represents the average of three separate independent experiments with the standard deviations for each point shown as error bars, where ** represents P < 0.05.


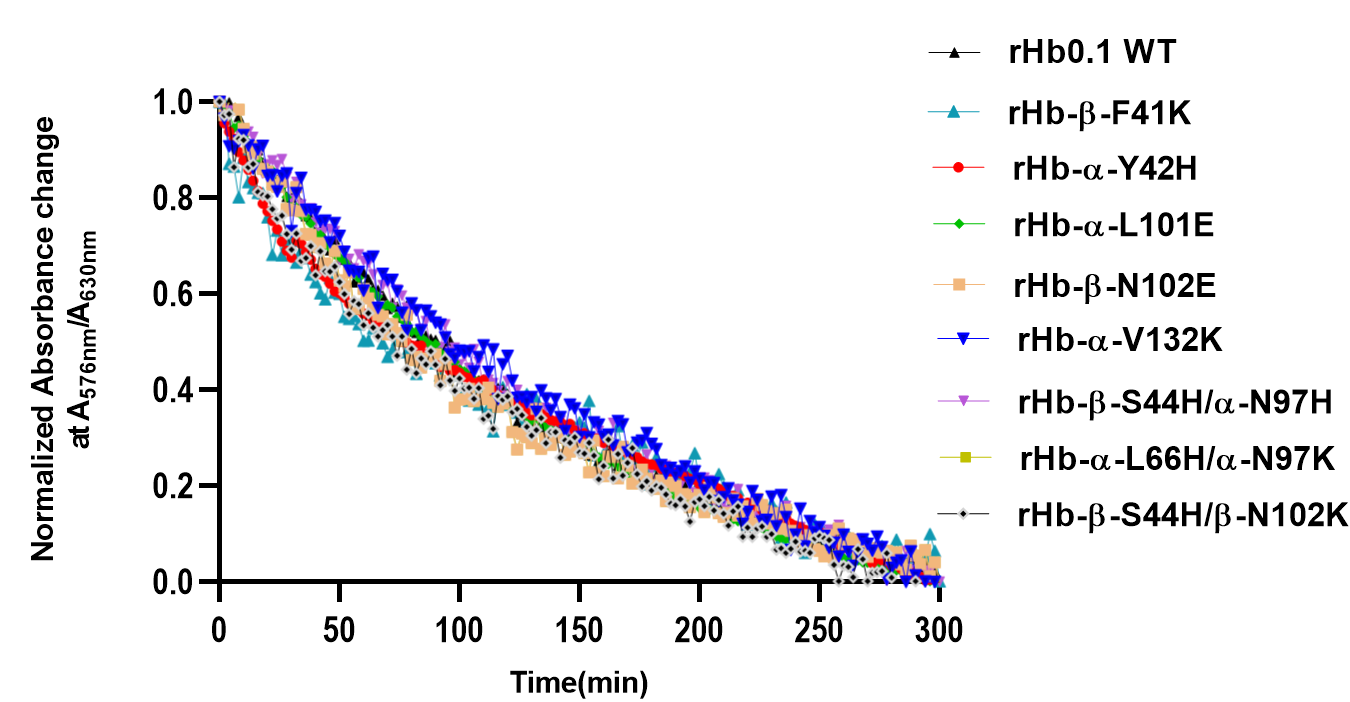


**Figure S2. Influence of mutations on auto-oxidation in hemoglobin.** Normalized absorbance time courses for the autooxidation of recombinant human hemoglobins at 25°C in air-equilibrated 0.1 M potassium phosphate, 1 mM EDTA, pH 7.5, containing 3 mmol/mol each of heme catalase and superoxide dismutase.

**Method S3**

**Stability studies to further validate the absence of covalent heme-protein cross link using UV-vis spectroscopy**

For GuHCl stability studies, the pure protein was diluted in various concentrations of GuHCl (Sisco Research Laboratories Pvt. Ltd, Maharashtra, India) (0–6.0 M) at pH 7.0 and incubated for 3–4 hours. The protein concentration used for stability studies was approximately 0.15 mg/ml. Absorbance spectra was then measured in the ranges of 260–700 nm. Data is an average of three independent experiments

**Result S3**

**Validation of Non-Covalent Heme-protein cross link in rHb-β-F41K through chemical denaturation studies**

The qualitative assessment of how heme pocket amino acid side chains affect heme retention capability, as described above, was further validated through spectroscopic investigations against denaturants. It is established that when a denaturant destabilizes hemoglobin (Hb), the distinct Soret peak experiences a shift toward shorter wavelengths (blue shift) along with a notable concurrent reduction in absorbance intensity. In case of complete heme dissociation from a globin, a broad absorbance peak characteristic of free heme emerges, with a Soret wavelength maximum as low as 350-370 nm, accompanied by a substantial decrease in intensity (27). To compare protein stability, we performed GuHCl-induced unfolding studies using absorbance spectroscopy. The Soret band of native HbA and rHb0.1 WT decreased on increasing the concentration of GuHCl, and a shoulder peak appeared at ∼370 nm, characteristic of free heme. This observation was similar to that of rHb-β-F41K, suggesting that heme was released upon unfolding in the absence of a covalent Lys–heme cross-link (Supplementary Figure S3 A and B). The transition curves showed an apparent denaturation midpoint at approximatively 1.0 M GuHCl for rHb0.1 WT, 1.2 M for HbA whereas 1.4 M GuHCl for rHb-β-F41K, indicating better globin stability of this mutant (Supplementary Figure S3 C).


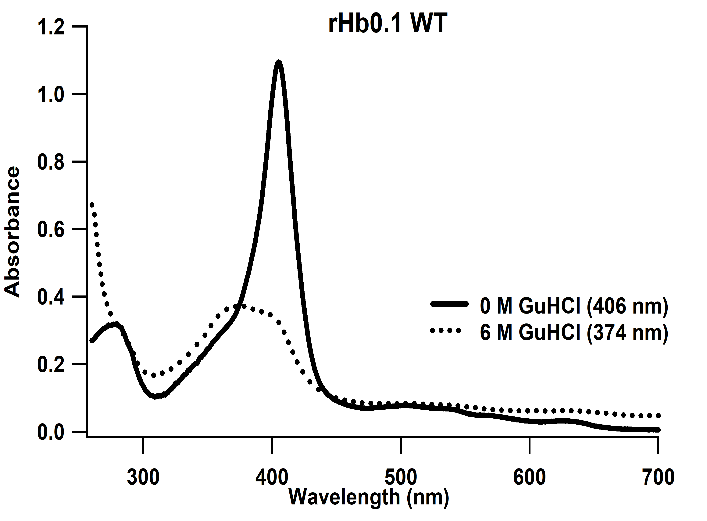

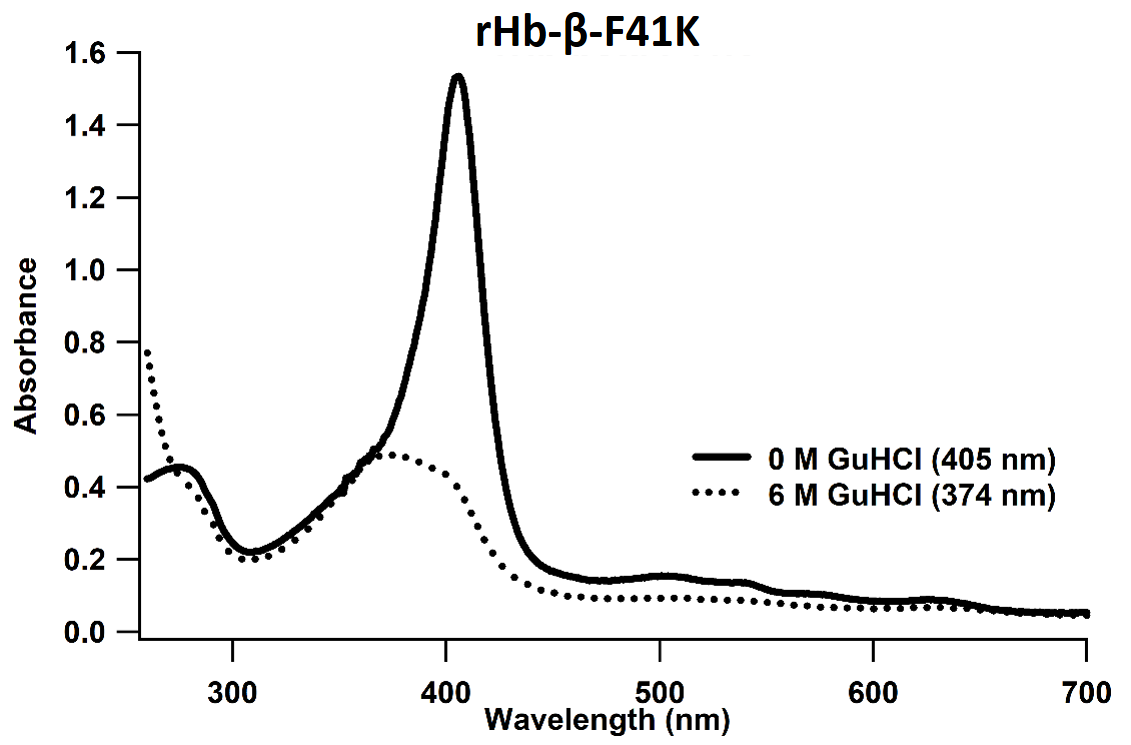


**(B)**

**(A)**

**(C)**


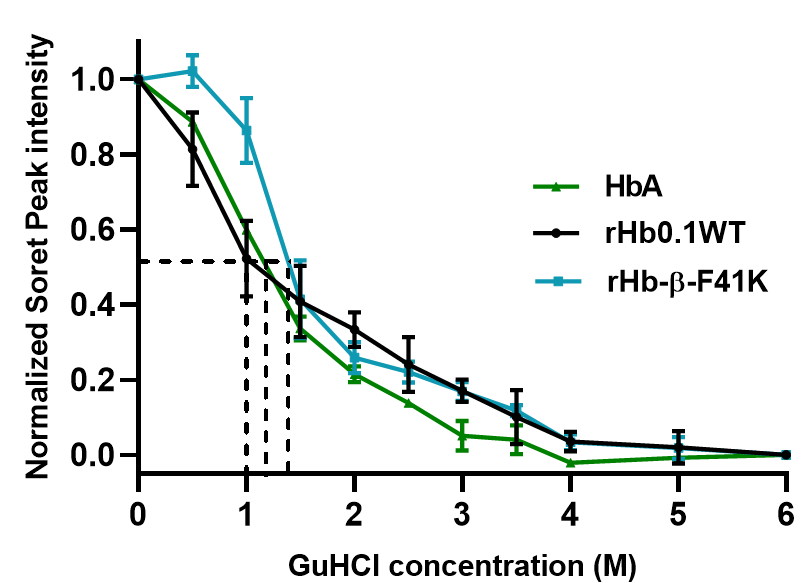


**Figure S3: GuHCl stability studies of recombinant hemoglobin 0.1 wild type (black) and mutant protein (blue).** (A) and (B) UV-vis spectral changes in proteins with increased GuHCl concentration monitored in the UV-vis range from 700-260 nm. (C) Changes in the Soret peak intensity were monitored with increases in GuHCl concentration demonstrating better apoglobin stability of β-F41K compared to rHb0.1WT and HbA (control protein). Three independent experiments were performed for this study.

**(B)**

**(A)**

**
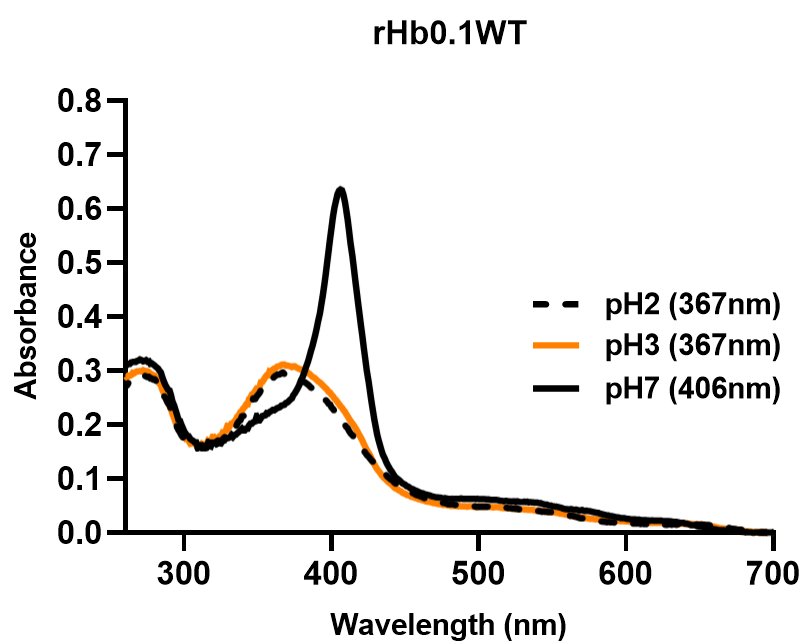
**  **
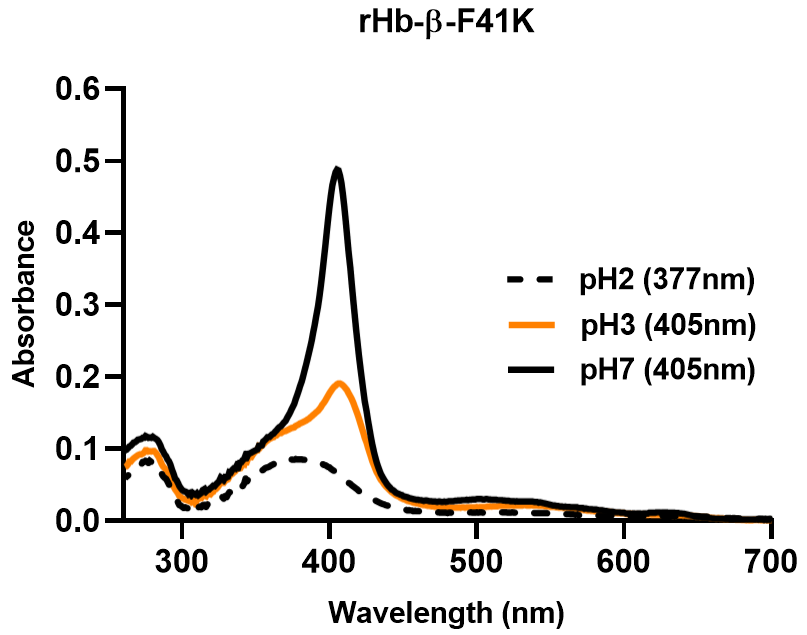
**

**(C)**


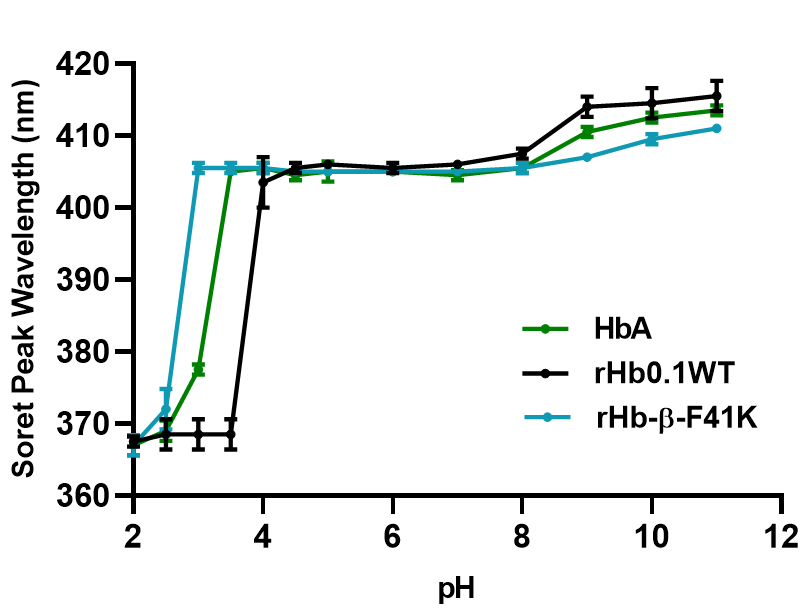


**Figure S4: pH stability studies of native HbA (control), recombinant hemoglobin 0.1 wild type and mutant protein β-F41K.** (A) and (B) Changes in UV-Vis absorption spectra of rHb0.1 WT and rHb--F41K incubated at pH range from 2.0 to 11.0 to assess holo-hemoglobin stability by monitoring Soret peak wavelength maxima and intensity. (C) Comparison of pH titration profile of HbA, rHb0.1 WT and mutant protein measured by monitoring Soret peak wavelength maxima, which shows that β-F41K can retain heme from pH 3.0-11.0 while rHb0.1 WT does the same from pH 4.0-11.0. Three independent experiments were performed for this study.

## Method S5

## Fluorescence spectroscopy. The intrinsic fluorescence emanating from the tryptophan residues within the recombinant Hb protein was assessed using an Eclipse Cary Varian UV-Vis spectrofluorometer (Varian Inc., Palo Alto, California, USA) coupled with a Peltier temperature controller. The protein present at a concentration of 0.15 mg/ml, was studied in a 100 mM phosphate buffer at pH 7.4. Spectra were collected spanning the 300-420 nm range, with an excitation wavelength of 292 nm and excitation and emission slits both set at 5 nm. The final spectrum was an average of three scans; subsequently each spectrum was subtracted from the blank spectra (27).

**Result S5**

Intrinsic tryptophan fluorescence spectroscopy is a reliable method for monitoring alterations in the immediate surroundings of aromatic amino acids. It offers insights into the naturally folded tertiary structure of the polypeptide (27). When we employed intrinsic fluorescence with specific excitation at 292 nm for both rHb0.1 WT and the representative mutant β-F41K, we observed emission peaks at 331 and 336 nm, respectively (Figure S5). This observation implies that the six tryptophan (Trp) residues are situated within the hydrophobic core of the densely packed protein molecule. However, the 5 nm shift in β-F41K with increased fluorescence intensity is due to the close vicinity of this residue to β-Trp37 which might cause the changes in the microenvironment of these fluorophores, including tryptophan shielding and positional shifts of tryptophan residues in the globin molecule. Consequently, we can confirm that all the rHb proteins have been purified while retaining its native, folded conformation, with the heme moiety securely bound (data not shown).


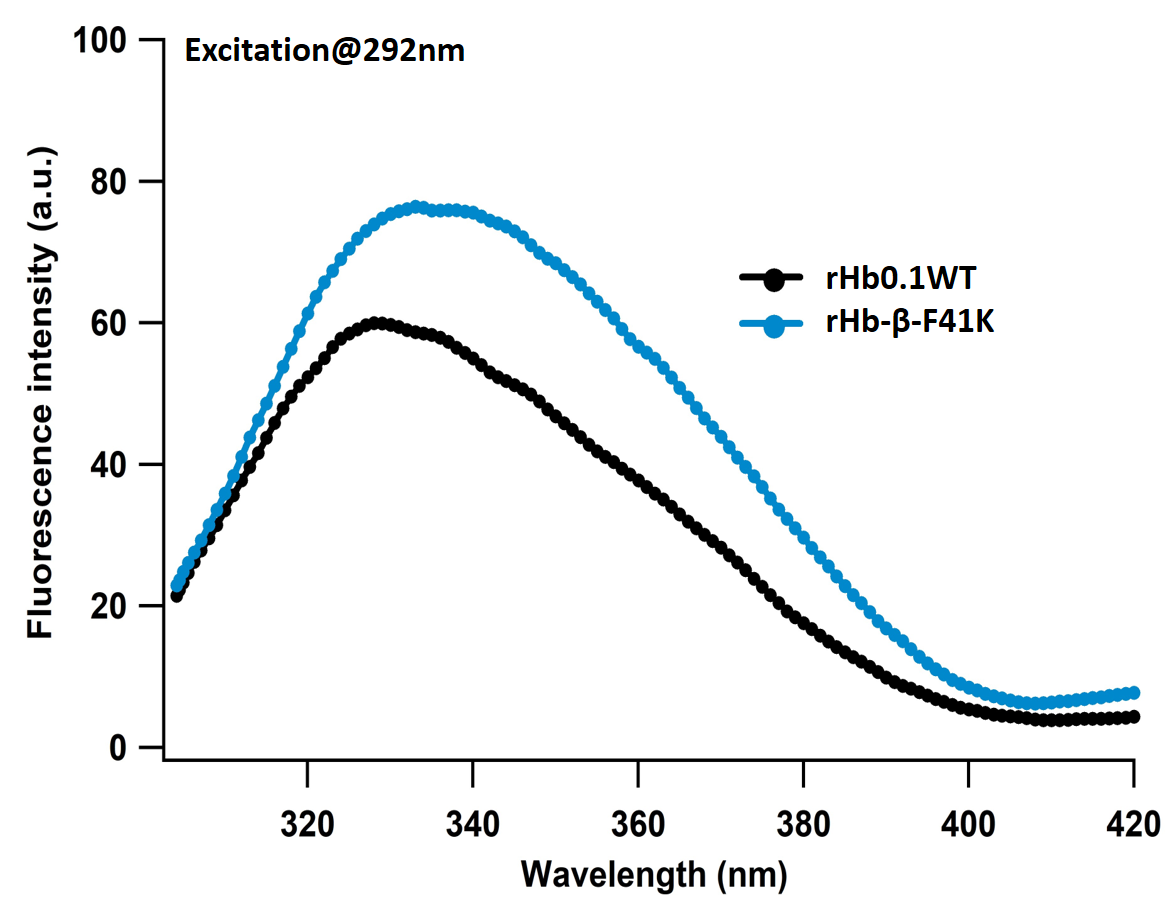


**Figure S5. Intrinsic tryptophan-specific fluorescence emission spectrum.** Recombinant human hemoglobin wild type and mutant rHb-β-F41K shows emission maxima at 331 and 336 nm, respectively, upon excitation at 292 nm, which indicates a non-polar buried environment for Trp residues in a natively folded protein.

**Method S6**

**Reverse phase chromatography**

Reverse phase chromatography was performed with 300 x 4.6 mm Kinetex C-18 (Phenomenex Inc., Torrance, USA) column on a Waters HPLC system (Waters, Milford, MA). Samples were analysed by injecting 50 µg of protein onto the column. The method involved two buffer solvents; solvent A containing 0.1% trifluoroacetic acid (Thomas Baker Chemicals Pvt. Ltd., Mumbai, India) in water and solvent B containing 0.1% TFA in acetonitrile (Thermo Fisher Scientific India Pvt. Ltd., Maharashtra, India). The flow rate of the mobile phase was set to 0.3 ml/min. The column was first equilibrated with solvent A for 5 mins. Protein was then eluted using a linear gradient of solvent B from 40 to 55% acetonitrile (containing 0.1% trifluoroacetic acid) for 40 mins. Then the solvent B was increased to 90% linearly for 3 mins for cleaning the column and then decreased to 10% over a 1 min interval. The spectra of Hb were observed at 280 nm. The integration and extraction of peaks were carried out utilizing Empower 2.0 software from Waters (Milford, MA, USA).

**Result S6**

**Reverse phase HPLC confirmed the absence of covalent linkage between heme and globin chains of mutant**

The non-covalent attachment of heme moiety to the protein matrix was further assessed by reverse-phase HPLC experiments. It was observed that almost the entire heme moiety from rHb0.1 wild type and its mutants dissociated and eluted as free heme at ~12.2 min, as also evidenced by the absence of any protein peak when monitored at 360 nm. The resulting apohemoglobin eluted between ~ 8-11 min as indicated by the measurement at 280 nm (Figure S6 A) [29]. The elution profile of Syn Hb showed a single peak at both 280 nm (blue line) and 360 nm (red line), indicating the presence of a covalently linked heme-protein complex. Since the sample was analysed on a C18 reverse-phase column using an acetonitrile gradient, the single peak suggested that the heme and protein were tightly bound, preventing the dissociation that might otherwise result in separate peaks for the protein (280 nm) and free heme (360 nm). Free heme absorbance spectrum shows the λ_max_ at 360 nm while the bound heme inside the protein matrix shows the Soret peak at 407 nm for Syn Hb (Figure S6 B).

(A)


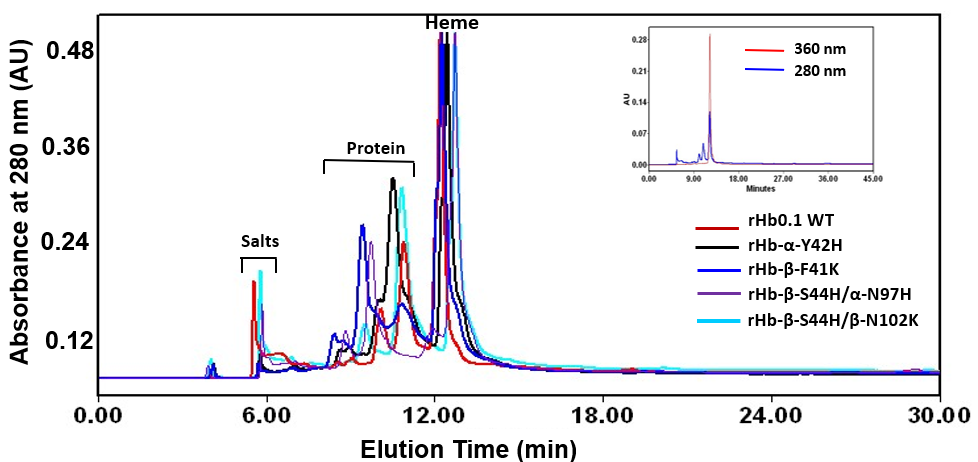


(B)


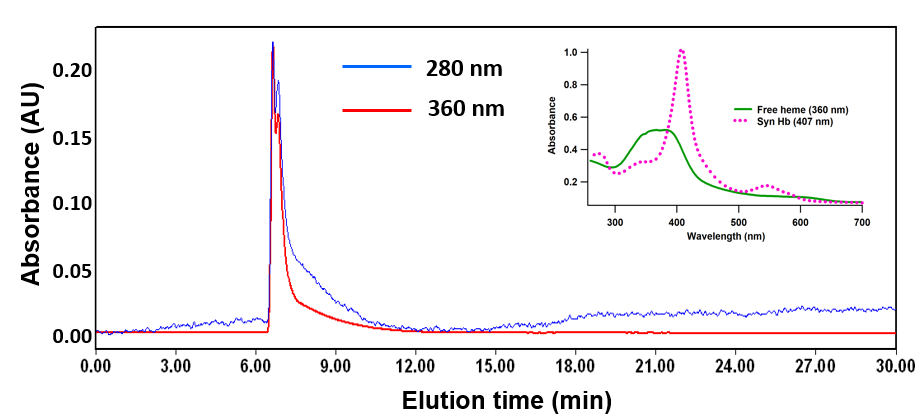


**Figure S6. Reversed phase HPLC analysis of purified rHb0.1 wild type and mutant proteins using Kinetex C18 column.** (A)The elution of the protein was detected at 280 nm. An acetonitrile gradient was used to determine the elution position of rHb0.1 wild type, rHb-α-Y42H, rHb-β-S44H/α-N97H, rHb-β-S44H/β-N102K and β-F41K proteins. Inset figure shows the elution of the free heme moiety determined at 360 nm, whereas the protein was monitored at 280 nm. This clearly indicates that heme is not covalently attached to the protein in any of the mutant. (B)The elution profile of SynHb shows a single peak at both 280 nm (blue line) and 360 nm (red line), indicating the presence of a covalently linked heme-protein complex. Inset figure shows the free heme absorbance spectra (λ_max_ = 360 nm) compared with protein spectra of Syn Hb (λ_max_ = 407 nm).


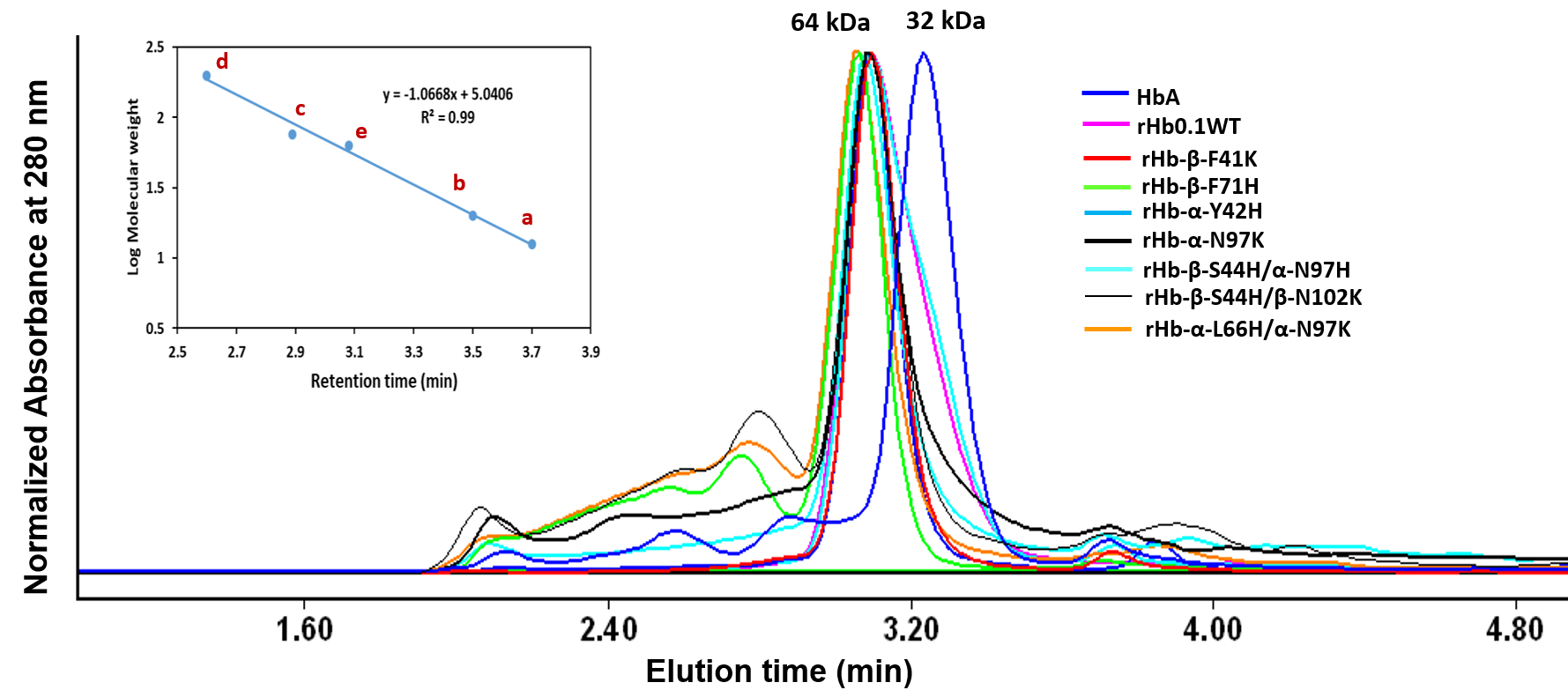


**Figure S7. Determination of the quaternary structure of recombinant hemoglobin mutants in solution**. HPLC analysis of rHb proteins on a size exclusion column at protein concentrations of 3 µM (e), with a, b, c, d and e depicting standard proteins (Lysozyme: 14.3 kDa, Trypsin: 24 kDa, Conalbumin: 76 kDa, Catalase: 240 kDa, and rHb0.1WT: 64 kDa, respectively) of known molecular weights. HbA showed dissociation of tetramer unlike rHb mutants having faster (α-N97K, β-F71H) and slower (β-F41K, α-Y42H, β-S44H/α-N97H, β-S44H/β-N102K, and α-L66H/α-N97K) heme dissociation kinetics than rHb0.1WT at the minimal concentration used for investigating heme dissociation kinetics.
